# Supplementary material for: Perceptual-cognitive skills and talent development environments in soccer: A scoping review
Source: PLoS One. 2025 Jul 8;20(7):e0327721. doi: 10.1371/journal.pone.0327721 (PMC12237070; doi:10.1371/journal.pone.0327721)
Supplement: Attachment — Submitted filename: PONE-D-24-10341_reviewer (1)3004.pdf [file pone.0327721.s002.pdf]

PLOS ONE

Perceptual-cognitive skills and talent identification in soccer: A scoping review of the last 20 years

--Manuscript Draft--

**Manuscript Number:**	PONE-D-24-10341
**Article Type:**	Research Article
**Full Title:**	Perceptual-cognitive skills and talent identification in soccer: A scoping review of the last 20 years
**Short Title:**	Scoping review of perceptual cognitive skills in soccer
**Corresponding Author:**	Andrew Triggs, MSc<br>UCFB College of Football Business Ltd<br>Manchester, UNITED KINGDOM
**Keywords:**	Visual-Search; Anticipation; Decision-Making; performance; Football; Coaches; Scouts
**Abstract:**	Perceptual-cognitive skills are a key component for successful soccer performance. Over the years, youth soccer academies have a looked to identify and develop perceptual cognitive skills to improve the quality of players in their talent development environment. While there is a wealth of interest in perceptual cognitive skills in soccer, the research associating these skills to the talent identification and development process is relatively limited. The present study provides a scoping review, examining perceptual cognitive skills within soccer-specific environments over the last twenty years, with a particular focus on talent identification and development contexts. Following PRISMA guidelines, specific inclusion and exclusion criteria were set, where forty-eight studies were included in the final review. Analysis identified key themes in the soccer-specific perceptual cognitive literature, including visual search behaviours; cognitive capabilities; performance; methodologies; and stakeholders. These themes are discussed with areas for future research identified to support the work of stakeholders in talent identification and development contexts (e.g., coaches, scouts, academy directors), further bridging the gap between science and application.
**Order of Authors:**	Andrew O. Triggs, MSc
	Joe Causer, PhD
	Allistair P. McRobert, PhD
	Matthew Andrew, PhD
**Additional Information:**	
**Question**	**Response**
**Financial Disclosure**<br><br>Enter a financial disclosure statement that describes the sources of funding for the work included in this submission. Review the submission guidelines for detailed requirements. View published research articles from *PLOS ONE* for specific examples.<br><br>This statement is required for submission and **will appear in the published article** if the submission is accepted. Please make sure it is accurate.	The author(s) received no specific funding for this work.

**Funded studies**<br>Enter a statement with the following details:<br>• Initials of the authors who received each award<br>• Grant numbers awarded to each author<br>• The full name of each funder<br>• URL of each funder website<br>• Did the sponsors or funders play any role in the study design, data collection and analysis, decision to publish, or preparation of the manuscript?<br><br>Did you receive funding for this work?	
**Competing Interests**<br><br>Use the instructions below to enter a competing interest statement for this submission. On behalf of all authors, disclose any competing interests that could be perceived to bias this work—acknowledging all financial support and any other relevant financial or non-financial competing interests.<br><br>This statement is required for submission and **will appear in the published article** if the submission is accepted. Please make sure it is accurate and that any funding sources listed in your Funding Information later in the submission form are also declared in your Financial Disclosure statement.<br><br>View published research articles from *PLOS ONE* for specific examples.	The authors have declared that no competing interests exist.

Powered by Editorial Manager® and ProduXion Manager® from Aries Systems Corporation

**NO authors have competing interests**<br><br>Enter: *The authors have declared that no competing interests exist.*<br><br>**Authors with competing interests**<br><br>Enter competing interest details beginning with this statement:<br><br>*I have read the journal's policy and the authors of this manuscript have the following competing interests: [insert competing interests here]*<br><br>* typeset	
**Ethics Statement**<br><br>Enter an ethics statement for this submission. This statement is required if the study involved:<br><br>• Human participants<br>• Human specimens or tissue<br>• Vertebrate animals or cephalopods<br>• Vertebrate embryos or tissues<br>• Field research<br><br>Write "N/A" if the submission does not require an ethics statement.<br><br>General guidance is provided below. Consult the submission guidelines for detailed instructions. **Make sure that all information entered here is included in the Methods section of the manuscript.**	N/A

**Format for specific study types**<br><br>**Human Subject Research (involving human participants and/or tissue)**<br>• Give the name of the institutional review board or ethics committee that approved the study<br>• Include the approval number and/or a statement indicating approval of this research<br>• Indicate the form of consent obtained (written/oral) or the reason that consent was not obtained (e.g. the data were analyzed anonymously)<br><br>**Animal Research (involving vertebrate animals, embryos or tissues)**<br>• Provide the name of the Institutional Animal Care and Use Committee (IACUC) or other relevant ethics board that reviewed the study protocol, and indicate whether they approved this research or granted a formal waiver of ethical approval<br>• Include an approval number if one was obtained<br>• If the study involved *non-human primates*, add *additional details* about animal welfare and steps taken to ameliorate suffering<br>• If anesthesia, euthanasia, or any kind of animal sacrifice is part of the study, include briefly which substances and/or methods were applied<br><br>**Field Research**<br><br>Include the following details if this study involves the collection of plant, animal, or other materials from a natural setting:<br>• Field permit number<br>• Name of the institution or relevant body that granted permission	
**Data Availability**<br><br>Authors are required to make all data underlying the findings described fully available, without restriction, and from the time of publication. PLOS allows rare exceptions to address legal and ethical concerns. See the PLOS Data Policy and FAQ for detailed information.	Yes - all data are fully available without restriction

A Data Availability Statement describing where the data can be found is required at submission. Your answers to this question constitute the Data Availability Statement and **will be published in the article**, if accepted.<br><br>**Important:** Stating 'data available on request from the author' is not sufficient. If your data are only available upon request, select 'No' for the first question and explain your exceptional situation in the text box.<br><br>Do the authors confirm that all data underlying the findings described in their manuscript are fully available without restriction?	
**Describe where the data may be found in full sentences. If you are copying our sample text, replace any instances of XXX with the appropriate details.**<br><br>• If the data are **held or will be held in a public repository**, include URLs, accession numbers or DOIs. If this information will only be available after acceptance, indicate this by ticking the box below. For example: *All XXX files are available from the XXX database (accession number(s) XXX, XXX.).*<br>• If the data are all contained **within the manuscript and/or Supporting Information files**, enter the following: *All relevant data are within the manuscript and its Supporting Information files.*<br>• If neither of these applies but you are able to provide **details of access elsewhere**, with or without limitations, please do so. For example:<br><br>*Data cannot be shared publicly because of [XXX]. Data are available from the XXX Institutional Data Access / Ethics Committee (contact via XXX) for researchers who meet the criteria for access to confidential data.*<br><br>*The data underlying the results presented in the study are available from (include the name of the third party*	All relevant data are within the manuscript and its Supporting Information files.

and contact information or URL).<br>• This text is appropriate if the data are owned by a third party and authors do not have permission to share the data.<br><br>* typeset	
Additional data availability information:	Tick here if the URLs/accession numbers/DOIs will be available only after acceptance of the manuscript for publication so that we can ensure their inclusion before publication.

Manuscript
Click here to access/download;Manuscript;25.03.2024
PLOSOnePCSandTIDinSoccerReviewManuscript.docx

Perceptual-cognitive skills and talent identification in soccer: A scoping review of the last 20 years

Andrew O. Triggs[1, 2*], Joe Causer[2], Allistair P. McRobert[2], and Matthew Andrew[3]

[1]UCFB Manchester Campus, Manchester, UK

[2]Research Institute for Sport and Exercise Sciences
Liverpool John Moores University, Liverpool, UK

[3]Department for Sport and Exercise Science,
Manchester Metropolitan University Institute of Sport, Manchester, UK

[*]Corresponding author
UCFB Manchester Campus,
14th Floor, 111 Piccadilly, Manchester, M1 2HY, UK
Tel: +44 161 247 2000; Email: a.o.triggs@2021.ljmu.ac.uk

Abstract

Perceptual-cognitive skills are a key component for successful soccer performance. Over the years, youth soccer academies have a looked to identify and develop perceptual cognitive skills to improve the quality of players in their talent development environment. While there is a wealth of interest in perceptual cognitive skills in soccer, the research associating these skills to the talent identification and development process is relatively limited. The present study provides a scoping review, examining perceptual cognitive skills within soccer-specific environments over the last twenty years, with a particular focus on talent identification and development contexts. Following PRISMA guidelines, specific inclusion and exclusion criteria were set, where forty-eight studies were included in the final review. Analysis identified key themes in the soccer-specific perceptual cognitive literature, including visual search behaviours; cognitive capabilities; performance; methodologies; and stakeholders. These themes are discussed with areas for future research identified to support the work of stakeholders in talent identification and development contexts (e.g., coaches, scouts, academy directors), further bridging the gap between science and application.

Keywords: Visual-Search; Anticipation; Decision-Making; Performance; Football; Coaches; Scouts

Introduction

The process of identifying talented youth soccer players that have the potential to develop into experts/professionals in adulthood and contribute to their club's future success is the aim of many soccer academies and nations (Reeves et al., 2018). Over recent years, professional soccer clubs have invested significantly in their talent identification (TID) and development (TDE) programmes by employing qualified coaches (Güllich et al., 2020), scouts (Roberts et al., 2019a), sport scientists (Bennett et al., 2019b) and performance analysts (Reeves & Roberts, 2013) to identify and develop youth players. These soccer academies aim to provide an environment that educates and inspires young players around the technical, tactical, physical, mental, lifestyle, and well-being aspects of life and soccer (Williams & Reilly, 2000). To support TID and TDE processes, many researchers have extensively examined some of the key predictors of future expert performance including physical (e.g., speed; Carling et al., 2009; Datson et al., 2020), skill (e.g., technical; Höner et al., 2017; Höner et al., 2019; Rechenchosky et al., 2021), sociological (e.g., developmental activities; Ford et al., 2020; Andrew et al., 2021), psychological (e.g., motivation; Höner & Feichtinger, 2016), and chance events (e.g., relative age effect; Andrew et al., 2022) (for a review, see Williams et al., 2020). This evidence-based information can be translated to professional soccer clubs to aid identification, development, and (de)selection of players (Reilly et al., 2000; Williams et al., 2020).

Physiological and anthropometrical attributes are widely researched in TID settings (Meylan et al., 2010) due to objectivity of measurement and observation practicality (Thelwell et al., 2006; Sullivan et al., 2023), as well as physical skills (e.g., speed endurance) typically being identified as fundamental skills for professional/senior soccer (Le Gall et al., 2010). This is complemented within the soccer industry, where the traditional, subjective nature of TID by stakeholders, and a lack of formal education has resulted in physical skills being the forefront of TID practices (Unnithan et al., 2012; Furley & Memmert, 2016; Sarmento et al., 2018). The lack of formal education has resulted in those responsible for TID often overlooking less visible skills that are also future key predictors of expert performance (Svensson & Drust, 2005; Dodd & Newans, 2018; Murr et al., 2018; Dugdale et al., 2020). Therefore, scouts/coaches often misconstrue anthropometrics as skill (Cripps et al., 2016) and therefore 'taller'

and ‘heavier’ players are viewed as more talented and thus are often selected/retained within soccer academies (Dugdale et al., 2021).

It is well accepted that in addition to advanced technical/motor skills (e.g., passing, dribbling), expert soccer players possess high levels of perceptual-cognitive skills (PCS; Williams et al., 2020). These skills are defined as an ability to identify and acquire environmental information that is integrated with existing knowledge, where an appropriate goal-directed response(s) can be selected and executed (Marteniuk, 1976). PCS are complex but include: *visual search*, which refers to an athletes ability to attend to important/relevant information of the unfolding event(s)/action(s) (Williams & Davids, 1998); *anticipation*, which refers to an athletes ability to predict the event(s)/action(s) that are likely to unfold prior to an event occurring (Williams & Jackson, 2019); and *decision-making*, which refers to an athletes ability to use information from the current situation to plan, select and execute an appropriate goal-directed action(s) (Memmert & Roca, 2019). PCS can allow soccer players to make faster and more effective decisions (Vaeyens et al., 2007a; Vítor de Assis et al., 2020), identify and utilise multiple sources of information efficiently (Vater et al., 2019), enhance creativity (Roca et al., 2018), and improve body positioning (Jordet et al., 2020). However, the work to date of TID and TDE in youth academy soccer continues to overlook the links between PCS and performance. Although there are clear links between PCS, soccer and TID/TDE, there has yet to be a review of this literature to examine the current understanding and identify gaps in knowledge.

Present study

The purpose of the current review is to examine literature surrounding PCS in soccer, detailing the manner these skills have been investigated, with the aim to ascertain areas of future research to provide further information for those responsible for TID and TDE (scouts/coaches), to be integrated within club’s processes. Due to the diverse literature base and methodological approaches, a scoping review was deemed the most appropriate strategy to map the literature, identify themes, and provide informed suggestions on future PCS research in TID/TDE contexts (Mays et al., 2001; Arksey & O’Malley, 2005; Higgins & Green, 2011). As outlined in **Figure 1**, there has been an acceleration in PCS research interest, following some of the initial engagement with soccer specific PCS research

(Williams et al., 1993). Thus, the current review focuses on research in the last two decades. To our knowledge, this is the first review to explore research surrounding PCS in soccer-specific environments.

*Insert **Figure 1** around here*

Methods

A scoping review was selected over a systematic review as: (1) the research area has not yet been extensively reviewed; (2) there is a diverse body of literature pertaining to a broad topic; (3) there are a greater range of study designs and methodologies within the literature examining PCS (Mays et al., 2001; Arksey & O'Malley, 2005; Higgins & Green, 2011). The methodological framework includes identifying the research question; identifying relevant studies; selecting relevant studies; charting the data; and summarising, collating the data and reporting the results (Arksey & O'Malley, 2005). Preferred Reporting Items for scoping reviews (PRISMA-ScR) checklist items were held to maintain methodological and reporting quality (Tricco et al., 2018). The principal research question was "what is the extent and nature of research conducted on PCS in soccer-specific environments, with research outcomes focussed on TID and TDE over the last twenty years?".

Search strategy

The search strategy was initiated in January 2024 and completed in February 2024. To ensure quality and appropriate coverage, the following electronic databases were used to search for relevant publications; PubMed, PsychInfo, SPORTDiscus, and Web of Science. Further records were searched for through Google Scholar, where additional relevant articles were hand-picked.

Search term one focused on mode of sport and was 'football' OR 'soccer'. Search term two focused on differing terms surrounding PCS (Mann et al., 2007; Broadbent et al., 2015) and was either 'perceptual-cognitive skills', 'game-intelligence', 'scan*', 'anticipation', 'decision-making', and 'visual-search'. Search term three 'coach*' was used in addition to the outcomes of search term one and two combined. This term was used to focus on TID/TDE processes, where coaches are one of the main stakeholders involved in such processes (Roberts et al., 2019a), and aimed to allow inclusion of articles which included applications of PCS findings. The asterisk used for 'scan*' and 'coach*' acted

as a truncation operator and allows variations of the key word to be found. Search term combinations that returned too many results with lack of specificity to the topic area were removed.

*Insert **Table 1** around here*

Screening process

The initial search revealed 2,348 results across core databases. Articles were title and key word screened for relevance independently by two reviewers (AT; MA) then screened by a third reviewer (JC). Articles meeting this initial inclusion criteria were downloaded and indexed using a bibliographic manager EndNoteX9 (Clarivate, UK) allowing effective management of the publications.

For the publications selected, duplicates were removed (*n* = 329) and abstract screening commenced (*n* = 543) independently by the same reviewers where the following inclusion criteria needed to be met: empirical articles; publications from the last twenty years (2003-2023); within soccer; focus had to be on PCS or key terms related to this; mention applications/implications within TID/TDE settings or direct applications to stakeholders in the field; participants were from a soccer-team-environment; the article published in English; and had to relate to outfield players/not include analysis of penalty kicks due to its lack of representation of the whole game of soccer.

*Insert **Table 2** around here*

Reviewers utilised the red-amber-green (RAG) rating scale due to its universal simplicity and understanding of the reviewer's status with their decision-making process (Robertson et al., 2017), where any amber rated publications were discussed with additional members of the research team. If a decision could not be made or the decision to include/exclude was not unanimous, the most experienced authors made the final decision (JC; AM). Data, recordings, and coding from articles were extracted into a single Microsoft Excel spreadsheet for validation and coding.

The articles remaining (n = 117) were full text screened by the same reviewers, utilising the same inclusion/exclusion criteria stated, before quality checks by two additional reviewers (JC; AM) (**Figure 2**.). Amber coded articles followed the same agreement processes as previous.

After coding, the remaining publications (n = 48) were appraised for: major foci of the study; participants and contexts; methodology; correlates and associations related to PCS in TID/TDE in soccer; main findings and major contribution to the literature; and application to TID/TDE or uses for stakeholders.

*Insert **Figure 2** around here*

Given the review focus, there were no clearly defined questions with focused parameters, nor were studies to be analysed regarding the quality of their methodological approach due to the variety and diversity of research encompassing PCS in soccer (Arksey & O'Malley, 2005). Moreover, a formal paper-by-paper critical quality assessment was not undertaken as this is not a key component of scoping reviews (Grant & Booth, 2009; Tricco et al., 2018). **Table 3** is an overview of the reviewed publications.

*Insert **Table 3** around here*

Results and discussion

The aim of the review was to analyse PCS in soccer and the links with TID and TDE, where emerging themes and sub-themes identified from the analysis (**Table 4**) were appraised, alongside providing proposals for future research to support the literature and practitioners (Reeves et al., 2018).

*Insert **Table 4** around here*

Visual search behaviours

Scanning

Visual search behaviour comprises of scan frequency, scan excursion, and fixation locations (Roca et al., 2018). 30 of the 48 studies examined visual search behaviour through varying terminology (gaze strategies, scanning, visual attention, exploration activity). Scanning, as a visual search behaviour has typically been measured through observational methods (Phatak & Gruber, 2019; McGuckian et al., 2018; 2020; Jordet et al., 2020; Aksum et al., 2021a; Pokolm et al., 2022; Caso et al., 2023), due to the simplicity, cost effectiveness, and ecological validity (Gilmartin-Thomas et al., 2018), providing insights into the frequency and locations of visual search behaviours of professional/elite performers whilst being able to link them more specifically to performance variable outcomes (Mann et al., 2007). Visual search analysis from live competition shows relationships between scanning frequency and location prior to receiving the ball and positive outcomes such as forward movements, body orientation, successful and more impactful passes, and turns out of pressure (McGuckian et al., 2018; 2019; 2020; Aksum et al., 2021a; 2021b; Pokolm et al., 2022; Caso et al., 2023). Furthermore, contextual variables such as playing position, pitch location, possession status, pressure on player-in-possession, time remaining, as well as team situation (winning, losing, drawing), impact scanning behaviours (Jordet et al., 2020; Aksum et al., 2021a). This provides the truest representation of visual search behaviours because of the environment players are exercising such skills (Larkin & O'Connor, 2017; McGuckian et al., 2018; 2020). It is crucial to continue research in these contexts in combination with eye tracking methodologies, as Aksum et al., (2021b) highlights that during 11 vs. 11 in-situ games do not include many fixations at all (2.3%) when scanning, when there is a whole body of research highlighting the importance of fixation locations (e.g., Vaeyens et al., 2007a; Savelsbergh et al., 2010; Bishop et al., 2014).

Participants in observational studies include players from the European Championships (Phatak & Gruber, 2019), U17-U19 European Championships (Pokolm et al., 2022), English Premier League (EPL; Jordet et al., 2020), Eredivisie Dutch League (Caso et al., 2023), English (U17-U19; Aksum et al., 2021a), and Australian Youth academies (U15-U17; McGuckian et al., 2020), as well as semi-professional teams (McGuckian et al., 2018). This broad range of levels has provided an understanding of scanning behaviours across the soccer landscape during in-game scenarios. Although this research provides benefits to understanding PCS being exercised in 'natural' environments at high levels, greater

focus on visual search behaviours in TDE contexts is required. Youth development phases (YDP; U15-U17), merging into the professional development phase (PDP; U18s) abilities, closer to replicating the performance environment have been prioritised. Evaluating the same behaviours in academy contexts within the foundation phase (FP; U9-U11) and early YDP (U12-U14) where PCS development is crucial from a learning perspective (Winkler, 2001). This data will provide a stronger understanding of academy soccer players visual search development in relation to the abilities identified at the top of the sport, helping to guide practitioners working in TID/TDE environments. By providing information on U9-U14 players skills and contrasting with professional players, practitioners can work with players to develop scan frequency and excursion to improve performance outcomes (Janacsek et al., 2012; Jordet et al., 2020).

Fixation behaviours

In addition to scanning behaviours, fixation locations pre, during and post action-execution are crucial to understanding soccer player's visual search capabilities (Fransen et al., 2017). Analysis of visual function tests across youth age groups indicated that soccer players 'hardware' (e.g., visual acuity) did not differentiate skill levels at any age (Ward & Williams, 2003) began the trajectory of this research area. Development of eye tracking technology provides fixation locations, durations, and gaze strategies in differing moments (Savelsbergh et al., 2002). Representative task designs are often utilised whereby several key behavioural mechanisms have been determined (Savelsbergh et al., 2010; Roca et al., 2013; Bishop et al., 2014; Vítor de Assis et al., 2020). Typically, in-game temporal occlusion tasks (static or live images) where participants are required to select an option they predict to occur and/or decide the best option to take (e.g., pass, dribble, shoot) responding via button press (Bishop et al., 2014), movements to spaces (Savelsbergh et al., 2010), passing actions (Vaeyens et al., 2007a), or verbal reports (Roca et al., 2021). In approaches utilising representative designs, time to first fixate on the ball and leg area have been shown to underpin successful anticipation and decision-making skills (Savelsbergh et al., 2010; Bishop et al., 2014). In offensive scenarios, fixating on the player-in-possession improves reaction times in low number overloaded attacking scenarios (2 vs. 1), yet when numbers increase (5 vs. 3) fixating on teammates who are closely marked improves reaction time and accuracy of decision (Vaeyens et al., 2007a). This pattern continues in 11 vs. 11 scenarios where players

with high anticipation skills used significantly more fixations of shorter duration and toward greater number of locations (Roca et al., 2013; Vítor de Assis et al., 2020). Furthermore, highly creative players also employ a broader attentional focus towards more informative locations and in a different sequential order, whilst also detecting teammates in threatening positions earlier in the attacking scenario (Roca et al., 2018; 2021). There is a greater understanding of positional differences in visual search behaviours where there is a key theme that more fixations of shorter duration can improve anticipation and decision-making processes (Vítor de Assis et al., 2021; Dambroz et al., 2022; Ballet et al., 2023),), and can differentiate between age (Machado et al., 2017), ability (Natasuhara et al., 2022), decision-making systems (Cardoso et al., 2023) and is influenced by high physical workload (Casanova et al., 2022)

The isolation of key visual search metrics in such methodological designs is the dominant approach for fixation behaviour analysis. Current research identifying the relationship between the physical scanning behaviours and the fixation behaviours is currently limited; moving towards an integrated and holistic understanding of all the key visual search behaviours, progressing from the work of Aksum et al., (2021b) would provide the next steps to appreciating the demands and requirements of an expert soccer player and allowing TDE practitioners to understand what can be done to support the development of such abilities (Roca & Williams, 2016).

Peripheral vision

Peripheral vision detects information in the visual field outside the limits of central/foveal vision (≈99 % visual field; Magill, 2007; Rosenholtz, 2016). With game dynamics, scanning behaviours cannot always occur (Araújo et al., 2006; Williams & Jackson, 2019). Verbal reports (verbalised mental thoughts) following 3 vs. 3 scenarios indicates highly skilled soccer players actively monitor opposition and teammates actions via peripheral vision (Vater et al., 2019). While interest on the importance of developing peripheral vision in soccer is increasing (e.g., Schumacher et al., 2019; 2020) there is limited literature support. The focus remains on what players can see and interpret within their direct field of view. Further understanding how TDE contexts can support the development of peripheral vision training (e.g., Junior, 2018) and identification through effective practice designs and would be valuable to advance these under trained skills (Schumacher et al., 2020).

Perceptual-cognitive skills and cognitive capabilities

Decision-making and anticipation

Decision-making has been subdivided into accuracy and speed (Helsen & Pauwels, 1993; Vaeyens et al, 2007b; Van Maarseveen et al., 2018), where speed is impacted by anticipatory abilities (Williams & Jackson, 2019). Professional soccer players make faster and a greater number of decisions both in and out of possession (Teoldo et al., 2023). At youth levels players exhibit different decision-making speeds both between and within ages at academy level (Andrade et al., 2021), but no differences in accuracy between the same ages (Farahani et al., 2020), where those exhibiting higher technical skills make more accurate decisions within ages (Vítor de Assis et al., 2020), and is not determined by successful anticipation (Roca et al., 2013). Video-based decision-making tasks can discriminate levels of expertise for youth athletes (Keller et al., 2018), where Machado et al., (2023a) suggests there are differences in decision-making quality and time for those selected and non-selected in youth systems (Machado et al., 2023a), highlighting the importance of understanding decision-making behaviours. Integrating environmental constraints (i.e., time; pressure) to examine the impact of decision-making capabilities is required to provide further context to our understanding (Musculus et al., 2021). This in turn, will support TDE environments where coaches can develop aspects such as practice design and coaching interventions to improve decision-making development (Práxedes et al., 2018; Coutinho et al., 2023; Machado et al., 2023b).

There is now progression towards an integrated approach, where the research field is uncovering the relationships and interactions between different PCS components; Anticipation and decision-making efficiency are impacted by saccade (ballistic eye movement between fixations) times to the ball as well as visual search strategies (Roca et al., 2013; Bishop et al., 2014), where higher skilled players utilise more fixations of shorter duration in a different sequential order and to more task-relevant locations of the display (Vaeyens et al., 2007a; Roca & Ford, 2020; Roca et al., 2021). Identifying if TID/TDE stakeholders' perceptions of the links between visual search capabilities, anticipation, and decision-making exist would allow researchers to determine if these findings are translated to the applied field (Broadbent et al., 2015).

Perception-action coupling

Pairing cognition with motor-execution in an applied soccer environment is crucial for coaches designing sessions to facilitate improvements in motor performance (Broadbent et al., 2015). Currently, the research has focused on decision-making and anticipation in isolation (e.g., Andrade et al., 2012; Beavan & Fransen, 2021; Bishop et al., 2014; Farahani et al., 2020; Roca et al., 2013), where a greater link between stimulus recognition speed, accuracy, and decision-making speed, as well as motor-execution, as the antecedents to the action is required to achieve a more accurate perception-action coupling appreciation (Farrow & Abernethy, 2003). More information of whether enhanced PCS improves speed, weight, and accuracy of the pass, change of direction, the distance a player gains from their opposition when receiving a pass, or the number of touches required prior a successful shot on goal are examples of the more in-depth information surrounding PCS execution that future research may need to explore in order to increase practitioner (i.e., coaches) buy-in.

Studies using representative task designs have added value to bridging the gap to applied practice, however, unless there is a comprehensive understanding of perception-action coupling within the applied environment, TID and TDE processes cannot be optimised (Williams et al., 2011). For example, if coaches were to fully understand the benefits of PCS during in-game scenarios (where players skills are examined in enjoinments that match the demands of the game) and how they contribute to soccer expertise, they can then integrate activities within their practice to develop PCS, and thus transfer of science to application would improve (Broadbent et al., 2015; Serra-Olivares et al., 2015; Gonçalves et al., 2017; Levi & Jackson, 2018). Retrospective verbal reports and ‘think aloud’ strategies (Roca et al., 2013; Eccles & Arsal, 2017) could support perception-action coupling, affording a window into the cognitive processes underpinning motor-execution in applied environments (McPherson & Thomas, 1989; Williams & Ericsson, 2005).

Performance

Game applications

Successful soccer performance is often associated with various outcomes such as successful passes, goals scored, tackles won, and distance covered etc. (Ric et al., 2017; Coutinho et al., 2018; Moalla et al., 2018). Visual scanning behaviours and the relationship with these performance metrics in 11 vs. 11 game formats has bridged this gap the furthest so far (Aksum et al., 2021a). However, the

term ‘performance’ branches out and has been examined in multiple contexts in the PCS domain within soccer such as passing performance following augmented feedback (Hicheur et al., 2020), 2 vs. 2 performance following pressure training interventions (Kent et al., 2021), eye function performance (Ward & Williams, 2003), decision-making performance based on visual search strategies in differing contextual environments (Vaeyens et al., 2007a; Vítor de Assis et al., 2020), and creative performance mechanisms utilising representative task designs (Roca et al., 2018; 2021). Performance in a holistic sense, and understanding what performance looks like during competition is an area which requires further exploration; there is a difference between test performance and performance in soccer. To promote TID/TDE practitioners engagement with research informed practice, it is crucial to demonstrate the significance of enhanced PCS in the game of soccer (Rosalie & Mueller, 2012).

Skill execution

The *Expert Performance Approach* aims to understand how expert performers acquire/refine skills across different sports and other domains (Ericsson & Smith, 1991). Currently the PCS literature has only examined differences between high and low performers and the mechanisms that underpin these differences due to the acceptance of differences in motor capabilities, thus there are likely differences in their PCS (Abernethy et al., 1993; Del Villar et al., 2004; 2007). Currently there is no literature that has examined any key developmental milestones of PCS expertise, it therefore becomes difficult to identify what mechanisms develop, at what rates and in which order that enable individuals to become experts in adulthood. Using a combination of both laboratory- and field-based approaches would be advantageous to gain a greater understanding of how soccer players PCS performance is impacted, and what could be done from an applied perspective to develop such mechanisms. It is noteworthy that there remains an inconsistency in classifying a ‘skilled’ (particularly high and low) or ‘elite’ athlete, which will ultimately shape the conclusions made if only examining either ends of the performance spectrum (McAuley et al., 2022).

Methodologies: Research designs

Task designs

Representative task designs using video and image-based occlusion combined with interaction methodologies have allowed for a greater understanding of how to develop PCS knowledge in soccer

(Ford et al., 2009). Simulating perception-action coupling via game-based scenarios, where with the support of high technology equipment, allows standardised and repeatable conditions, has facilitated greater insights into the mechanisms that underpin successful PCS in soccer (Starkes & Ericsson, 2003; Pinder et al., 2011). Sacrificing ecological validity but gaining more reliable data currently remains the focus, though current task designs do not accurately provide situations to replicate observations during *in situ* tasks (Broadbent et al., 2015). There was no correlation between *in situ* performance values provided by coaches over multiple 3 vs. 3 scenarios to measures of anticipation, decision-making, and pattern recall measured during the 'typical' task designs (van Maarseveen et al., 2018), highlighting the need to develop *in situ* measures, which to studies remain relatively limited (e.g., McGuckian et al., 2018; 2020; Jordet et al., 2020; Aksum et al., 2021a).

Technological advancements

Technological advancements have the ability to further bridge the gap between science and application within TID/TDE contexts, where practitioners can find a greater balance between subjective (Lund & Söderström, 2017) and objective information. For example, mobile eye tracking technology has enhanced our understanding of gaze and scanning strategies, allowing a significant distinguishment between high- and low-skilled athletes (Discombe & Cotterill, 2015; Moran et al., 2018). The aim is to utilise technological advancements to achieve a better understanding of what the athlete experiences, reads and responds to in a game of soccer, where the intensity is higher and the athlete's approach to the game is different due psychological factors (e.g., motivation, anxiety etc.) that will be interacting and exercised simultaneously (Krustrup et al., 2005; Bloomfield et al., 2007; Nédélec et al., 2012; Walsh, 2014). Aksum et al., (2021b) provided the most significant integration of eye tracking technology within live settings to date, collecting data from 4 players using eye tracking glasses during 11 vs. 11 matches . Similar work should be developed in other contexts. In doing so, understanding of fixation behaviours in athletes would be advanced significantly by improving data quantities to improve conclusions made.

Research within virtual reality (VR), has provided progress, where speed, decisions and actions are more representative than before whilst providing greater reliability and the feeling of immersion within a real-life experience (Pagé et al., 2019; Fortes et al., 2021). Though not perfect due to extraneous

variables and inconsistencies of contextual variables the game of soccer provides, it is a positive step (Romeas et al., 2019). For example, Soccer Bot 360^0 technology facilitates greater game representation, with 360-degree environments with multiple task formats and ranging stimuli (Kittel et al., 2020). However, it does not allow the user to influence the situation being presented in any way, decoupling perception and action (Pinder et al., 2011). As this technology is novel, there is a rationale to explore within TID/TDE settings to identify if, and how it can support practitioners. Clearly there is a balance to be achieved to ensure reliability and validity of measurements taken, where there will be a trade off in any research scenario.

Methodologies: Sporting context and participants

Coaches

Coaches are central to TID/TDE processes, with their knowledge, abilities and behaviours significantly impacting their players (Cushion et al., 2012; Andrew et al., 2024). Yet, there is little work to date with those responsible for identifying and developing PCS. Coaches' conceptualisations of decision-making, visual exploratory activity and creativity (Leso et al., 2017; O'Connor et al., 2018; Eldridge et al., 2023) is becoming clearer, alongside understanding coaching approaches in professional academies across Europe, where academies spend more practice time (via engagement in an educational workshop if needed) in activities that have an element of active decision-making (Roca & Ford, 2020; Andrew et al., 2022; Jones et al., 2023). Although spending time in such practices, coaches are not currently optimising coaching styles and interventions to elicit such abilities (O'Connor et al., 2017; 2018; Beavan & Fransen, 2021). This is impacted by perceptions, experience, and coaching qualification, which influences engagement in visual exploratory activities in coaches' sessions (Pulling et al., 2018; Eldridge et al., 2023). This research provides a positive but limited direction towards coaches' perceptions of certain PCS and how they can be developed within practice with a strong emphasis on decision-making. The importance in the identification, monitoring, and development of PCS in athletes should not be underestimated for coaches as they are the individuals who need to obtain sport specific knowledge in which they translate to multiple stakeholders effectively (Macdonald & Tinning, 1995; Cushion et al., 2003). Analysing how coaches' perceptions link to the practices currently being delivered to identify the relationship between knowledge and action would be beneficial.

Furthermore, understanding how coaches identify and monitor PCS could further improve such approaches in applied settings as if coaches cannot identify PCS, they will likely be unable to develop them (Williams & Ericsson, 2005; Schumacher et al., 2018).

Scouts

The soccer scout's primary role is to identify 'talented' youth players and can be very difficult (Bergkamp et al., 2019). The TID process is traditional and scouts must remove their (un)conscious biases regarding physical attributes and be able to identify, measure and record PCS within their assessments of future potential soccer performance of a player, which does not necessarily present as clear, observable behaviours (Johansson & Fahlén, 2017; Roberts et al., 2019b). Currently there is limited understanding of scouts' knowledge, perceptions, and identification of PCS. Highlighting these perceptions and how scouts are identifying and measuring such skills would further support practitioners. In addition, examining to what extent scouts are still being influenced by chance events (e.g., relative age effect; Andrew et al., 2022) or physical skills (Hill & Sotiriadou, 2016), or whether due to improved awareness and understanding of different future performance predictors (Bergkamp et al., 2022), their priorities have levelled. Also, whilst we have information on what skills scouts/coaches value during the TID process (Fuhre et al., 2022), it is currently unclear how this translates to practice. Mobile eye tracking has the potential to provide insights into the visual search strategies (as well as verbal reports) that underpin scouts and/or coach decisions to (de)select players within a youth soccer academy. Furthermore, assessment tools for decision-making capabilities are being tested, but with further developments to be made before they can fully support scouts in their TID processes (Bennett et al., 2019; de Joode et al., 2021).

Players

Research in soccer academies has been completed within highly skilled players. Previous studies examining PCS in elite soccer players have recorded data from players within EPL academies to explore areas of PCS expertise (Ward & Williams, 2003; Farahani et al., 2020; Kent et al., 2021). Whether there are differences in youth academies further down the soccer pyramid in England would be key to understand if PCS are a substantial differentiating factor of players in the top tier academies

from a TID perspective, or if there are differences in developmental process from those at the top of the pyramid versus further down.

Female soccer

Soccer continues to be one of the most popular female sports worldwide (FIFA, 2022), with the popularity rising exponentially (i.e., live/tv attendance; professionalisation etc.; Culvin & Bowes, 2023). Consequently, professional female soccer clubs and nations are advancing their TID and TDE processes. For example, in England, professional female clubs are now advancing their talent pathways similar to, and in line with, the men's pathways, with new structures providing up to 70 Emerging Talent Centres (ETC) allowing 95 per cent of players accessing an ETC within one hour of where they live by 2024 and the number of young female players engaged in FA talent programmes across the country rise from 1,722 to more than 4,200 by the end of the 2023-24 season (FA, 2023). Although scientific literature to support these processes are gradually rising (Kryger et al., 2021), they are still mostly examined in male soccer. For example, from studies examining TID between 1999-2019, 14 per cent of these studies were female only participants (Curran et al., 2019). Therefore, calls for more female-specific research have been made (Williams et al., 2020; Peters et al., 2022). PCS knowledge in female soccer players would be crucial, where there is an emerging body of research utilising female participants (van Maarseveen et al., 2018; Meira et al., 2022; Machado et al., 2023b). Research and TID/TDE processes from male players cannot be extrapolated to the female game, as they may (or may not) be erroneous (Emmonds et al., 2019), such as previously from a technical perspective (Barfield et al., 2002). If the technical components of female athletes differ, it is important to investigate with the same curiosity towards the PCS components of female athletes.

Limitations and future directions

This review is not without its limitations. Firstly, we only included English language, peer-reviewed studies. Excluding non-English studies may influence sample characteristics (e.g., location) and lead to the omission of potential correlates that may be of cultural significance (Küttel & Larsen, 2020). Yet, some evidence suggests that limiting our search to studies published in English may not always be ample to impact review findings (Morrison et al., 2012). Furthermore, a general weakness of scoping reviews concerns the lack of quality assessment of the included studies (Küttel & Larsen, 2020).

Reiterating recommendations from previous sections there are several areas for potential future examinations of PCS in soccer, particularly in TID/TDE contexts. These considerations emerged as a direct consequence of the results in this scoping review and focus primarily on research aims to be explored. In addressing each of these considerations, a more holistic and detailed understanding of PCS in soccer could be achieved, including: (1) integrate key stakeholders within TID/TDE contexts and understand how they identify, monitor, and evaluate PCS; (2) bridge the gap between representative tasks designs with field work, examining behaviour in real life scenarios; (3) include developmental monitoring of PCS in TID/TDE contexts; (4) examine the interaction between differing PCS further and how weaknesses in one area may impact another PCS; and (5) gain a holistic understanding of PCS abilities in all soccer player groups, including the women's game and all academy age categories.

Conclusion

The current scoping review aimed to provide original contributions to the body of research, delivering an overview of the contemporary work in soccer-specific contexts of PCS research. By thematically synthesising findings from a wide array of studies in this field, we aim to re-focus the direction of potential future research to support the growth of PCS identification and development, in youth athletes. Although there has been a significant shift in focus to this area of player development within the past twenty years, where the understanding of PCS and the differences between elite and non-elite athletes is strong; there remains notable gaps. The goal is to successfully identify PCS, understand the underlying mechanisms, and then how these can be impacted through applied practice. PCS expertise is a vital component of elite soccer performance (Williams et al., 2020) and thus the more evidence-based information, the higher the quality of player the talent development system can produce.

Funding details

This work was not associated with any funding agency or grant awarding body.

Disclosure statement

The authors report there are no competing interests to declare.

References

Abernethy B, Thomas KT, Thomas JT. Strategies for improving understanding of motor expertise [or mistakes we have made and things we have learned!!]. InAdvances in psychology 1993 Jan 1 (Vol. 102, pp. 317-356). North-Holland.

*Aksum KM, Pokolm M, Bjørndal CT, Rein R, Memmert D, Jordet G. Scanning activity in elite youth football players. Journal of Sports Sciences. 2021a Nov 2;39(21):2401-10.*A

*Aksum KM, Brotangen L, Bjørndal CT, Magnaguagno L, Jordet G. Scanning activity of elite football players in 11 vs. 11 match play: An eye-tracking analysis on the duration and visual information of scanning. Plos one. 2021b Aug 20;16(8):e0244118.

*Andrade L, Machado G, Gonçalves E, Teoldo I. Decision making in soccer: effect of positional role of U-13 soccer players. Journal of Physical Education and Sport. 2021 May 1;21(3):1413-20.

Andrew M, Baptiste GZ, Reeves MJ, Roberts SJ, McRobert AP, Ford PR. The developmental activities of skilled youth CONCACAF soccer players and the contribution of their development system. International Journal of Sports Science & Coaching. 2022 Dec;17(6):1363-77.

Andrew M, Finnegan L, Datson N, Dugdale JH. Men are from quartile one, women are from? Relative age effect in European soccer and the influence of age, success, and playing status. Children. 2022 Nov 14;9(11):1747.

Araújo D, Davids K, Hristovski R. The ecological dynamics of decision making in sport. Psychology of sport and exercise. 2006 Nov 1;7(6):653-76.

Arksey H, O'malley L. Scoping studies: towards a methodological framework. International journal of social research methodology. 2005 Feb 1;8(1):19-32.

*Ballet C, Barreto J, Hope E, Casanova F. What is the visual behaviour and attentional effort of football players in different positions during a real 11v11 game? A pilot study. F1000Research. 2023;12.

Barfield WR, Kirkendall DT, Yu B. Kinematic instep kicking differences between elite female and male soccer players. Journal of sports science & medicine. 2002 Sep;1(3):72.

*Beavan A, Fransen J. The influence of coaching instructions on decision-making in soccer. J. Sport Exerc. Sci. 2021;5:3-12.

*Bennett KJ, Novak AR, Pluss MA, Coutts AJ, Fransen J. Assessing the validity of a video-based decision-making assessment for talent identification in youth soccer. Journal of science and medicine in sport. 2019a Jun 1;22(6):729-34.

Bennett KJ, Vaeyens R, Fransen J. Creating a framework for talent identification and development in emerging football nations. Science and Medicine in Football. 2019b Jan 2;3(1):36-42.

Bergkamp TL, Frencken WG, Niessen AS, Meijer RR, den Hartigh RJ. How soccer scouts identify talented players. European Journal of Sport Science. 2022 Jul 3;22(7):994-1004.

Bergkamp TL, Niessen AS, den Hartigh RJ, Frencken WG, Meijer RR. Methodological issues in soccer talent identification research. Sports Medicine. 2019 Sep 1;49:1317-35.

*Bishop D, Kuhn G, Maton C. Telling people where to look in a soccer-based decision task: A nomothetic approach. Journal of eye movement research. 2014 Mar 19;7(2).

Bloomfield J, Polman R, O'Donoghue P. Physical demands of different positions in FA Premier League soccer. Journal of sports science & medicine. 2007 Mar;6(1):63.

Broadbent DP, Causer J, Williams AM, Ford PR. Perceptual-cognitive skill training and its transfer to expert performance in the field: Future research directions. European journal of sport science. 2015 May 19;15(4):322-31.

*Cardoso FD, Afonso J, Roca A, Teoldo I. The association between perceptual-cognitive processes and response time in decision making in young soccer players. Journal of Sports Sciences. 2021 Apr 18;39(8):926-35.

Carling C, Le Gall F, Reilly T, Williams AM. Do anthropometric and fitness characteristics vary according to birth date distribution in elite youth academy soccer players?. Scandinavian journal of medicine & science in sports. 2009 Feb;19(1):3-9.

*Casanova F, Esteves PT, Padilha MB, Ribeiro J, Williams AM, Garganta J. The effects of physiological demands on visual search behaviours during 2 vs. 1+ GK game situations in football: An in-situ approach. Frontiers in Psychology. 2022 May 30;13:885765.

*Caso S, Van Der Kamp Jo, Morel P, Savelsbergh G. The relationship between amount and timing of visual exploratory activity and performance of elite soccer players. Int. J. Sport Psychol. 2023;54:287-304.

*Coutinho D, Kelly AL, Santos S, Figueiredo P, Pizarro D, Travassos B. Exploring the Effects of Tasks with Different Decision-Making Levels on Ball Control, Passing Performance, and External Load in Youth Football. Children. 2023 Jan 26;10(2):220.

Coutinho D, Gonçalves B, Wong DP, Travassos B, Coutts AJ, Sampaio J. Exploring the effects of mental and muscular fatigue in soccer players' performance. Human movement science. 2018 Apr 1;58:287-96.

Cripps AJ, Hopper LS, Joyce C. Coaches' perceptions of long-term potential are biased by maturational variation. International journal of sports science & coaching. 2016 Aug;11(4):478-81.

Culvin A, Bowes A, editors. Women's football in a global, professional era. Emerald Publishing Limited; 2023 Mar 9.

Curran O, MacNamara A, Passmore D. What about the girls? Exploring the gender data gap in talent development. Frontiers in sports and active living. 2019 Jul 11;1:3.

Cushion C, Ford PR, Williams AM. Coach behaviours and practice structures in youth soccer: Implications for talent development. Journal of sports sciences. 2012 Nov 1;30(15):1631-41.

Cushion CJ, Armour KM, Jones RL. Coach education and continuing professional development: Experience and learning to coach. Quest. 2003 Aug 1;55(3):215-30.

*Dambroz F, Cardoso F, Neves JA, Teoldo I. Visual search strategies of young soccer players according to positional role. Motricidade. 2022 Jun 30;18(2):177-82.

Datson N, Weston M, Drust B, Gregson W, Lolli L. High-intensity endurance capacity assessment as a tool for talent identification in elite youth female soccer. Journal of Sports Sciences. 2020 Jun 17;38(11-12):1313-9.

*de Joode T, Tebbes DJ, Savelsbergh GJ. Game insight skills as a predictor of talent for youth soccer players. Frontiers in Sports and Active Living. 2021 Jan 15;2:609112.

Del Villar F, González LG, Iglesias D, Moreno MP, Cervelló EM. Expert-novice differences in cognitive and execution skills during tennis competition. Perceptual and motor skills. 2007 Apr;104(2):355-65.

Del Villar F, Iglesias D, Moreno MP, Fuentes JP, Cervelló EM. An investigation into procedural knowledge and decision-making: Spanish experienced-inexperienced basketball players differences. Journal of Human Movement Studies. 2004 Jan 1;46:407-20.

Discombe RM, Cotterill ST. Eye tracking in sport: A guide for new and aspiring researchers. Sport & Exercise Psychology Review. 2015 Sep;11(2):49-58.

Dodd KD, Newans TJ. Talent identification for soccer: Physiological aspects. Journal of science and medicine in sport. 2018 Oct 1;21(10):1073-8.

Dugdale JH, McRobert AP, Unnithan VB. Selected, deselected, and reselected: A case study analysis of attributes associated with player reselection following closure of a youth soccer academy. Frontiers in Sports and Active Living. 2021 Mar 23;3:633124.

Dugdale JH, Sanders D, Myers T, Williams AM, Hunter AM. A case study comparison of objective and subjective evaluation methods of physical qualities in youth soccer players. Journal of Sports Sciences. 2020 Jun 17;38(11-12):1304-12.

Eccles DW, Arsal G. The think aloud method: what is it and how do I use it?. Qualitative Research in Sport, Exercise and Health. 2017 Aug 8;9(4):514-31.

*Eldridge D, Pocock C, Pulling C, Kearney P, Dicks M. Visual exploratory activity and practice design: Perceptions of experienced coaches in professional football academies. International Journal of Sports Science & Coaching. 2023 Apr;18(2):370-81.

Emmonds S, Heyward O, Jones B. The challenge of applying and undertaking research in female sport. Sports medicine-open. 2019 Dec;5:1-4.

Ericsson KA, Smith J, editors. Toward a general theory of expertise: Prospects and limits. Cambridge University Press; 1991 Aug 30.

*Farahani J, Soltani P, Rezlescu C. Assessing decision-making in elite academy footballers using real-world video clips. Progress in brain research. 2020 Jan 1;253:59-70.

Farrow D, Abernethy B. Do expertise and the degree of perception—action coupling affect natural anticipatory performance?. Perception. 2003 Sep;32(9):1127-39.

FIFA. Setting the Pace. FIFA Benchmarking Report Women's Football. 2022. Available from https://digitalhub.fifa.com/m/70a3f8fbc383b284/original/FIFA-Benchmarking-Report-Womens-Football-Setting-the-pace-2022_EN.pdf

Ford P, Coughlan E, Williams M. The expert-performance approach as a framework for understanding and enhancing coaching performance, expertise and learning. International Journal of Sports Science & Coaching. 2009 Sep;4(3):451-63.

Ford PR, Hodges NJ, Broadbent D, O'Connor D, Scott D, Datson N, Andersson HA, Williams AM. The developmental and professional activities of female international soccer players from five high-performing nations. Journal of Sports Sciences. 2020 Jun 17;38(11-12):1432-40.

Fortes LS, Almeida SS, Praça GM, Nascimento-Júnior JR, Lima-Junior D, Barbosa BT, Ferreira ME. Virtual reality promotes greater improvements than video-stimulation screen on perceptual-cognitive skills in young soccer athletes. Human Movement Science. 2021 Oct 1;79:102856

*Fransen J, Lovell TW, Bennett KJ, Deprez D, Deconinck FJ, Lenoir M, Coutts AJ. The influence of restricted visual feedback on dribbling performance in youth soccer players. Motor control. 2017 Apr 1;21(2):158-67.

Fuhre J, Øygard A, Sæther SA. Coaches' criteria for talent identification of youth male soccer players. Sports. 2022 Feb;10(2):14.

Furley P, Memmert D. Coaches' implicit associations between size and giftedness: implications for the relative age effect. Journal of sports sciences. 2016 Mar 3;34(5):459-66.

Gilmartin-Thomas JF, Liew D, Hopper I. Observational studies and their utility for practice. Australian prescriber. 2018 Jun;41(3):82.

Gonçalves B, Esteves P, Folgado H, Ric A, Torrents C, Sampaio J. Effects of pitch area-restrictions on tactical behavior, physical, and physiological performances in soccer large-sided games. The Journal of Strength & Conditioning Research. 2017 Sep 1;31(9):2398-408.

Grant MJ, Booth A. A typology of reviews: an analysis of 14 review types and associated methodologies. Health information & libraries journal. 2009 Jun;26(2):91-108.

Güllich A, Cronauer R, Diehl J, Gard L, Miller C. Coach-assessed skill learning progress of youth soccer players correlates with earlier childhood practice in other sports. International Journal of Sports Science & Coaching. 2020 Jun;15(3):285-96.

Helsen W, Pauwels JM. The relationship between expertise and visual information processing in sport. InAdvances in psychology 1993 Jan 1 (Vol. 102, pp. 109-134). North-Holland.

*Hicheur H, Chauvin A, Cavin V, Fuchslocher J, Tschopp M, Taube W. Augmented-feedback training improves cognitive motor performance of soccer players. Medicine & Science in Sports & Exercise. 2020 Jan 1;52:141.

Hill B, Sotiriadou P. Coach decision-making and the relative age effect on talent selection in football. European sport management quarterly. 2016 May 26;16(3):292-315.

Höner O, Feichtinger P. Psychological talent predictors in early adolescence and their empirical relationship with current and future performance in soccer. Psychology of sport and exercise. 2016 Jul 1;25:17-26.

Höner O, Leyhr D, Kelava A. The influence of speed abilities and technical skills in early adolescence on adult success in soccer: A long-term prospective analysis using ANOVA and SEM approaches. PloS one. 2017 Aug 14;12(8):e0182211.

Höner O, Raabe J, Murr D, Leyhr D. Prognostic relevance of motor tests in elite girls' soccer: a five-year prospective cohort study within the German talent promotion program. Science and Medicine in Football. 2019 Oct 2;3(4):287-96.

Janacsek K, Fiser J, Nemeth D. The best time to acquire new skills: Age- related differences in implicit sequence learning across the human lif

Johansson A, Fahlén J. Simply the best, better than all the rest? Validity issues in selections in elite sport. International Journal of Sports Science & Coaching. 2017 Aug;12(4):470-80.

Jones B, Eather N, Miller A, Morgan PJ. Evaluating the impact of a coach development intervention for improving coaching practices and player outcomes in football: the MASTER Coaching randomised control trial. Physical Education and Sport Pedagogy. 2023 Nov 24:1-8.

*Jordet G, Aksum KM, Pedersen DN, Walvekar A, Trivedi A, McCall A, Ivarsson A. Scanning, contextual factors, and association with performance in English Premier League footballers: An investigation across a season. Frontiers in psychology. 2020 Oct 6;11:553813.

Junior NK. Peripheral vision training for the soccer: 10 years of the studies. MOJ Sports Med. 2018;2(5):133-5.

*Keller BS, Raynor AJ, Iredale F, Bruce L. Tactical skill in Australian youth soccer: Does it discriminate age-match skill levels?. International Journal of Sports Science & Coaching. 2018 Dec;13(6):1057-63.

*Kent S, Devonport TJ, Lane AM, Nicholls W. Implementing a pressure training program to improve decision-making and execution of skill among premier league academy soccer players. Journal of Applied Sport Psychology. 2022 Jul 4;34(4):691-712.

Kittel A, Larkin P, Cunningham I, Spittle M. 360 virtual reality: a SWOT analysis in comparison to virtual reality. Frontiers in Psychology. 2020 Oct 7;11:563474.

Krustrup P, Mohr M, Ellingsgaard HE, Bangsbo J. Physical demands during an elite female soccer game: importance of training status. Medicine & Science in Sports & Exercise. 2005 Jul 1;37(7):1242-8.

Küttel A, Larsen CH. Risk and protective factors for mental health in elite athletes: A scoping review. International Review of Sport and Exercise Psychology. 2020 Jan 1;13(1):231-65.

Larkin P, O'Connor D. Talent identification and recruitment in youth soccer: Recruiter's perceptions of the key attributes for player recruitment. PLOS one. 2017 Apr 18;12(4):e0175716

Le Gall F, Carling C, Williams M, Reilly T. Anthropometric and fitness characteristics of international, professional and amateur male graduate soccer players from an elite youth academy. Journal of science and medicine in sport. 2010 Jan 1;13(1):90-5.

*Leso G, Dias G, Ferreira JP, Gama J, Couceiro MS. Perception of creativity and game intelligence in soccer. Creativity Research Journal. 2017 Apr 3;29(2):182-7.

Levi HR, Jackson RC. Contextual factors influencing decision making: Perceptions of professional soccer players. Psychology of Sport and Exercise. 2018 Jul 1;37:19-25.

Lund S, Söderström T. To see or not to see: Talent identification in the Swedish Football Association. Sociology of Sport Journal. 2017 Sep 1;34(3):248-58.

Macdonald D, Tinning R. Physical education teacher education and the trend to proletarianization: A case study. Journal of teaching in physical education. 1995 Oct 1;15(1):98-118.

*Machado G, González-Víllora S, Teoldo I. Selected soccer players are quicker and better decision-makers in elite Brazilian youth academies. International Journal of Performance Analysis in Sport. 2023 Mar 4;23(2):65-82.

*Machado G, González-Víllora S, Teoldo I. The relationship between deliberate practice, play, and futsal in childhood and adolescence and the development of different decision-making skills in professional female soccer players. Psychology of sport and exercise. 2023 Sep 1;68:102470.

*Machado, G., Cardoso, F., & Teoldo, I. (2017) Visual search strategy of soccer players according to different age groups. Motriz: Revista de Educação Física, 23.

Magill, R. (2007). Touch, Proprioception, and Vision*. In *Motor Learning and Control. Concepts and Applications.* New York, NY: The McGraw-Hill Companies, 106–132.

Mann DT, Williams AM, Ward P, Janelle CM. Perceptual-cognitive expertise in sport: A meta-analysis. Journal of sport and exercise psychology. 2007 Aug 1;29(4):457-78.

Marteniuk RG. Cognitive information processes in motor short-term memory and movement production. InMotor control 1976 Jan 1 (pp. 175-186). Academic Press.

McAuley AB, Baker J, Kelly AL. Defining “elite” status in sport: from chaos to clarity. German Journal of Exercise and Sport Research. 2022 Mar;52(1):193-7.

*McGuckian TB, Cole MH, Chalkley D, Jordet G, Pepping GJ. Constraints on visual exploration of youth football players during 11v11 match-play: The influence of playing role, pitch position and phase of play. Journal of Sports Sciences. 2020 Mar 18;38(6):658-68.

*McGuckian TB, Cole MH, Chalkley D, Jordet G, Pepping GJ. Visual exploration when surrounded by affordances: frequency of head movements is predictive of response speed. Ecological Psychology. 2019 Jan 2;31(1):30-48.

*McGuckian TB, Cole MH, Jordet G, Chalkley D, Pepping GJ. Don’t turn blind! The relationship between exploration before ball possession and on-ball performance in association football. Frontiers in psychology. 2018 Dec 10;9:402968.

McPherson SL, Thomas JR. Relation of knowledge and performance in boys' tennis: Age and expertise. Journal of experimental child psychology. 1989 Oct 1;48(2):190-211.

*Meira C, Cortes C, Corbetta D, Oliveira D, Moreira SR, Silva B, Massa M. Gaze behaviour differentiates elite from non-elite female soccer players: a 2D video projections exploratory study. European Journal of Human Movement. 2022(49):9.

Memmert D, Roca A. Tactical creativity and decision making in sport. InAnticipation and decision making in sport 2019 Feb 12 (pp. 201-214). Routledge.

Meylan C, Cronin J, Oliver J, Hughes M. Talent identification in soccer: The role of maturity status on physical, physiological and technical characteristics. International Jour

Moalla W, Fessi MS, Makni E, Dellal A, Filetti C, Di Salvo V, Chamari K. Association of physical and technical activities with partial match status in a soccer professional team. The Journal of Strength & Conditioning Research. 2018 Jun 1;32(6):1708-14.

Moran A, Campbell M, Ranieri D. Implications of eye tracking technology for applied sport psychology. Journal of Sport Psychology in Action. 2018 Oct 2;9(4):249-59.

Morrison A, Polisena J, Husereau D, Moulton K, Clark M, Fiander M, Mierzwinski-Urban M, Clifford T, Hutton B, Rabb D. The effect of English-language restriction on systematic review-based meta-analyses: a systematic review of empirical studies. International journal of technology assessment in health care. 2012 Apr;28(2):138-44.

Murr D, Raabe J, Höner O. The prognostic value of physiological and physical characteristics in youth soccer: A systematic review. European journal of sport science. 2018 Jan 2;18(1):62-74.

*Musculus L, Bäder J, Sander L, Vogt T. The influence of environmental constraints in 360 videos on decision making in soccer. Journal of Sport and Exercise Psychology. 2021 Sep 1;43(5):365-74.

*Natsuhara T, Kato T, Nakayama M, Yoshida T, Sasaki R, Matsutake T, Asai T. Decision-making while passing and visual search strategy during ball receiving in team sport play. Perceptual and motor skills. 2020 Apr;127(2):468-89.

Nédélec M, McCall A, Carling C, Legall F, Berthoin S, Dupont G. Recovery in soccer: part I—post-match fatigue and time course of recovery. Sports medicine. 2012 Dec;42:997-1015.

*O'Connor D, Larkin P, Williams AM. What learning environments help improve decision-making?. Physical Education and Sport Pedagogy. 2017 Nov 2;22(6):647-60.

*O'Connor D, Wardak D, Goodyear P, Larkin P, Williams M. Conceptualising decision-making and its development: a phenomenographic analysis. Science and Medicine in Football. 2018 Oct 2;2(4):261-71.

Okholm Kryger K, Wang A, Mehta R, Impellizzeri FM, Massey A, McCall A. Research on women's football: a scoping review. Science and Medicine in Football. 2022 Dec 1;6(5):549-58.

Pagé C, Bernier PM, Trempe M. Using video simulations and virtual reality to improve decision-making skills in basketball. Journal of sports sciences. 2019 Nov 2;37(21):2403-10.

Peters CM, Hendry DT, Hodges NJ. A scoping review on developmental activities of girls' and women's sports. Frontiers in Sports and Active Living. 2022 Sep 23;4:903886.

*Phatak A, Gruber M. Keep your head up—correlation between visual exploration frequency, passing percentage and turnover rate in elite football midfielders. Sports. 2019 Jun 6;7(6):139.

Pinder RA, Davids K, Renshaw I, Araújo D. Representative learning design and functionality of research and practice in sport. Journal of Sport and Exercise Psychology. 2011 Feb 1;33(1):146-55.

Plan, E.P.P. Document prepared by the English premier League. Elite Player Performance Plan. 2011. Available from:https://www. goalreports. com/EPLPlan. pdf.

*Pokolm M, Rein R, Müller D, Nopp S, Kirchhain M, Aksum KM, Jordet G, Memmert D. Modeling players' scanning activity in football. Journal of Sport and Exercise Psychology. 2022 Apr 25;44(4):263-71.

*Práxedes A, Moreno A, Gil-Arias A, Claver F, Del Villar F. The effect of small-sided games with different levels of opposition on the tactical behaviour of young footballers with different levels of sport expertise. PloS one. 2018 Jan 10;13(1):e0190157.

*Pulling C, Kearney P, Eldridge D, Dicks M. Football coaches' perceptions of the introduction, delivery and evaluation of visual exploratory activity. Psychology of Sport and Exercise. 2018 Nov 1;39:81-9.

Rechenchosky L, Menegassi VM, Jaime MD, Borges PH, Sarmento H, Mancha-Triguero D, Serra-Olivares J, Rinaldi W. Scoping review of tests to assess tactical knowledge and tactical performance of young soccer players. Journal of sports sciences. 2021 Sep 17;39(18):2051-67.

Reeves MJ, Roberts SJ. Perceptions of performance analysis in elite youth football. International Journal of Performance Analysis in Sport. 2013 Apr 1;13(1):200-11.

Reeves MJ, McRobert AP, Littlewood MA, Roberts SJ. A scoping review of the potential sociological predictors of talent in junior-elite football: 2000–2016. Soccer & Society. 2018 Nov 17;19(8):1085-105.

Reilly T, Williams AM, Nevill A, Franks A. A multidisciplinary approach to talent identification in soccer. Journal of sports sciences. 2000 Jan 1;18(9):695-702.

Ric A, Torrents C, Gonçalves B, Torres-Ronda L, Sampaio J, Hristovski R. Dynamics of tactical behaviour in association football when manipulating players' space of interaction. PloS one. 2017 Jul 14;12(7):e0180773.

Roberts AH, Greenwood DA, Stanley M, Humberstone C, Iredale F, Raynor A. Coach knowledge in talent identification: A systematic review and meta-synthesis. Journal of science and medicine in sport. 2019 Oct 1;22(10):1163-72.

Roberts SJ, McRobert AP, Lewis CJ, Reeves MJ. Establishing consensus of position-specific predictors for elite youth soccer in England. Science and Medicine in Football. 2019 Jul 3;3(3):205-13.

Robertson S, Bartlett JD, Gastin PB. Red, amber, or green? Athlete monitoring in team sport: the need for decision-support systems. International journal of sports physiology and performance. 2017 Apr 1;12(s2):S2-73.

*Roca A, Ford PR. Decision-making practice during coaching sessions in elite youth football across European countries. Science and Medicine in Football. 2020 Oct 1;4(4):263-8.

Roca A, Williams AM. Expertise and the interaction between different perceptual-cognitive skills: Implications for testing and training. Frontiers in psychology. 2016 May 25;7:195864.

*Roca A, Ford PR, Memmert D. Creative decision making and visual search behavior in skilled soccer players. PloS one. 2018 Jul 10;13(7):e0199381.

*Roca A, Ford PR, Memmert D. Perceptual-cognitive processes underlying creative expert performance in soccer. Psychological Research. 2021 Apr;85(3):1146-55.

*Roca A, Ford PR, McRobert AP, Williams AM. Perceptual-cognitive skills and their interaction as a function of task constraints in soccer. Journal of Sport and Exercise Psychology. 2013 Apr 1;35(2):144-55.

Romeas, T., Chaumillon, R., Labbé, D. and Faubert, J., 2019. Combining 3D-MOT with sport decision-making for perceptual-cognitive training in virtual reality. *Perceptual and Motor Skills*, *126*(5), pp.922-948.

Rosenholtz R. Capabilities and limitations of peripheral vision. Annual review of vision science. 2016 Oct 14;2:437-57.

Sarmento H, Anguera MT, Pereira A, Araújo D. Talent identification and development in male football: A systematic review. Sports medicine. 2018 Apr;48:907-31.

*Savelsbergh GJ, Haans SH, Kooijman MK, van Kampen PM. A method to identify talent: Visual search and locomotion behavior in young football players. Human movement science. 2010 Oct 1;29(5):764-76.

Savelsbergh GJ, Williams AM, Kamp JV, Ward P. Visual search, anticipation and expertise in soccer goalkeepers. Journal of sports sciences. 2002 Jan 1;20(3):279-87.

Schumacher N, Reer R. On-field perceptual-cognitive training improves peripheral reaction in soccer: A controlled trial. Frontiers in psychology. 2020 Aug 7;11:562046.

Schumacher N, Schmidt M, Reer R, Braumann KM. Peripheral vision tests in sports: Training effects and reliability of peripheral perception test. International Journal of Environmental Research and Public Health. 2019 Dec;16(24):5001.

Schumacher N, Schmidt M, Wellmann K, Braumann KM. General perceptual-cognitive abilities: Age and position in soccer. PloS one. 2018 Aug 23;13(8):e0202627.

Serra-Olivares J, González-Víllora S, García-López LM, Araújo D. Game-based approaches' pedagogical principles: exploring task constraints in youth soccer. Journal of human kinetics. 2015 Jun 1;46(1):251-61.

Starkes JL, Ericsson KA. Expert performance in sports: Advances in research on sport expertise.

Sullivan J, Roberts SJ, Mckeown J, Littlewood M, McLaren-Towlson C, Andrew M, Enright K. Methods to predict the timing and status of biological maturation in male adolescent soccer players: A narrative systematic review. Plos one. 2023 Sep 8;18(9):e0286768.

Svensson M, Drust B. Testing soccer players. Journal of sports sciences. 2005 Jun 1;23(6):601-18.

*Teoldo I, Mezzadri E, Cardoso F, Machado G. Speed of decision-making as a key element for professional and academy soccer players' performances. Retos. 2023;50:1195-203.

The Football Association. Revamped women and girls' player pathway aims to discover a new generation. 2023. Available from: https://www.englandfootball.com/articles/2023/Feb/09/new-emerging-talent-centres-launched-as-womens-girls-talent-pathway-revamped 20230902#:~:text=The%20changes%20will%20see%2095,of%20the%202023%2D24%20season.

Thelwell RC, Greenlees IA, Weston NJ. Using psychological skills training to develop soccer performance. Journal of applied sport psychology. 2006 Sep 1;18(3):254-70.

Tricco AC, Lillie E, Zarin W, O'Brien KK, Colquhoun H, Levac D, Moher D, Peters MD, Horsley T, Weeks L, Hempel S. PRISMA extension for scoping reviews (PRISMA-ScR): checklist and explanation. Annals of internal medicine. 2018 Oct 2;169(7):467-73

Unnithan, V., White, J., Georgiou, A., Iga, J., & Drust, B. (2012) Talent identification in youth soccer. *Journal of sports sciences*, 30 (15), 1719-1726.

*Vaeyens R, Lenoir M, Williams AM, Mazyn L, Philippaerts RM. The effects of task constraints on visual search behavior and decision-making skill in youth soccer players. Journal of Sport and Exercise Psychology. 2007a Apr 1;29(2):147-69.

Vaeyens R, Lenoir M, Williams AM, Philippaerts RM. Mechanisms underpinning successful decision making in skilled youth soccer players: An analysis of visual search behaviors. Journal of motor behavior. 2007 Sep 1;39(5):395-408.

*van Maarseveen MJ, Oudejans RR, Mann DL, Savelsbergh GJ. Perceptual-cognitive skill and the in situ performance of soccer players. Quarterly journal of experimental psychology. 2018 Feb;71(2):455-70.

*Vater C, Luginbühl S, Magnaguagno L. Testing the functionality of peripheral vision in a mixed-methods football field study. Journal of sports sciences. 2019 Dec 17;37(24):2789-97.

*Vítor de Assis J, Costa V, Casanova F, Cardoso F, Teoldo I. Visual search strategy and anticipation in tactical behavior of young soccer players. Science and Medicine in Football. 2021 Apr 3;5(2):158-64.

*Vitor de Assis J, González-Víllora S, Clemente FM, Cardoso F, Teoldo I. Do youth soccer players with different tactical behaviour also perform differently in decision-making and visual search strategies?. International Journal of Performance Analysis in Sport. 2020 Nov 1;20(6):1143-56.

Walsh V. Is sport the brain’s biggest challenge?. Current biology. 2014 Sep 22;24(18):R859-60.

*Ward P, Williams AM. Perceptual and cognitive skill development in soccer: The multidimensional nature of expert performance. Journal of sport and exercise psychology. 2003 Mar 1;25(1):93-111

Williams AM, Davids K. Visual search strategy, selective attention, and expertise in soccer. Research quarterly for exercise and sport. 1998 Jun 1;69(2):111-28.

Williams AM, Ericsson KA. Perceptual-cognitive expertise in sport: Some considerations when applying the expert performance approach. Human movement science. 2005 Jun 1;24(3):283-307.

Williams AM, Ford PR. ‘Game intelligence’: anticipation and decision making. InScience and soccer 2013 Jan 4 (pp. 117-133). Routledge.

Williams AM, Jackson RC. Anticipation in sport: Fifty years on, what have we learned and what research still needs to be undertaken?. Psychology of Sport and Exercise. 2019 May 1;42:16-24.

Williams AM, Reilly T. Talent identification and development in soccer. Journal of sports sciences. 2000 Jan 1;18(9):657-67.

Williams AM, Ford PR, Drust B. Talent identification and development in soccer since the millennium. Journal of sports sciences. 2020 Jun 17;38(11-12):1199-210.

Williams AM, Ford PR, Eccles DW, Ward P. Perceptual- cognitive expertise in sport and its acquisition: Implications for applied cognitive psychology. Applied Cognitive Psychology. 2011 May;25(3):432-42.

Williams M, Davids K, Burwitz L, Williams J. Cognitive knowledge and soccer performance. Perceptual and Motor skills. 1993 Apr;76(2):579-93.

Winkler W. Motor skills and cognitive training for junior soccer players. International journal of performance analysis in sport. 2001 Jul 1;1(1):91-105

Tables

Table 1. Overview of search strategy with quantity of results returned.

Search Term 1	Search Term 2	Search Term 3	PubMed	SPORTDiscus	PsychInfo	Web of Science	Google Scholar
Soccer OR Football	Game Intelligence		5	20	4	17	976
Soccer OR Football	Game Intelligence	Coach*	1	10	3	9	711
Soccer OR Football	Scan*		Search Discarded	Search Discarded	Search Discarded	Search Discarded	Search Discarded
Soccer OR Football	Scan*	Coach*	9	47	30	46	17,200
Soccer OR Football	Anticipation		114	115	90	379	17,600
Soccer OR Football	Anticipation	Coach*	5	13	6	16	17,200
Soccer OR Football	Decision-making		Search Discarded	Search Discarded	Search Discarded	Search Discarded	Search Discarded
Soccer OR Football	Decision-making	Coach*	120	248	101	364	20,500
Soccer OR Football	Visual Search		45	53	40	194	9,790
Soccer OR Football	Visual Search	Coach*	4	6	4	24	3,790
Soccer Or Football	Perceptual-Cognitive Skills		31	60	25	62	7,590
Soccer Or Football	Perceptual-Cognitive Skills	Coach*	4	12	2	10	4,880

Table 2. Inclusion criteria.

Characteristic	Inclusion Criteria
Language (Eligibility Criteria 1)	English Speaking.
Study (Eligibility Criteria 2)	Empirical in nature.
Practical Implications (Eligibility Criteria 3)	Based on applied TID/TDE practices or specific applications provided for TID/TDE practitioner (i.e., coaches/scouts)
Participants (Eligibility Criteria 4)	Soccer players/coaches/scouts) from a team environment.
Area (Eligibility Criteria 5)	Perceptual cognitive skills (or related terms) specific to soccer.
Time (Eligibility Criteria 6)	Publications from the past twenty years (2003-2023).
Soccer Specificity (Eligibility Criteria 7)	Use of outfield players and does not utilise the analysis of penalty kicks

Table 3. Summary of reviewed articles that measured PCS in a TID/TDE contexts in soccer.

Authors	Study Aim	Study Sample	Method	Important Results/Findings
Aksum et al., 2021a	To examine how the scanning behaviour of elite youth soccer players in Europe related to players' performance, according to situational, context-specific, and temporal constraints.	Outfield players (N = 53) from the four teams who reached the finals in the 2018 UEFA European U17 (n = 24) and U19 (n = 29) Championships.	Data was collected by filming the semi-finals and finals of the 2018 UEFA European U17 and U19 Championships.	U19 players performed more scans than U17 players.<br><br>There is a positive relationship between scan frequency and pass success.<br><br>Opponent pressure and pitch position are critical contextual factors that influence scanning behaviour.<br><br>Central midfielders and central defenders were found to have higher scan frequencies.
Aksum et al., 2021b	To explore the scanning behaviour of four elite football midfield players in 11 vs. 11 match play.	Four male central midfield players aged 17 to 23 who played for two different clubs in the Norwegian Premier League.	Two 11 vs. 11 matches played: an internal training match and a local third division team. Participants wore eye-tracking devices. Two of the players were recorded for 20 minutes each, and two players were recorded for 10 minutes each.	Action undertaken with the ball at the moment of scanning initiation influenced scanning duration.<br><br>Playing phase and player-to-ball distance influenced the number of teammates and opponents inside the video frame during scanning.<br><br>Very few scans involved fixations.<br><br>Scanning duration is not influenced by player-to-ball distance and playing phase.
Andrade et al., 2021	To verify the influence of positional role on the decision-making skills of U-13 soccer players.	30, U-13 male soccer players, from regional soccer schools (Brazil).	TacticUP® online platform used to measure decision-making quality and speed.	No differences in decision-making quality between the different positional roles.<br><br>Midfielders made quicker decisions, when compared to defenders and forwards, in situations near and distant from the ball.

				Positional role influenced the decision-making time of U-13 soccer players.
Ballet et al., 2023	To explore the visual behaviour and attentional effort of three football players in specific-role positions in the five seconds before receiving the ball.	A Left Back, Centre Midfielder and Right Winger male football players who play as amateurs in the Portugal National University Championships	11v11 game was used, where 24 game sequences were recorded and analysed with the Tobii Pro eye-movement registration glasses.	Players acting in different specific positions presented distinctive visual behaviours and attentional effort during a football game.
Beavan & Fransen, 2021	To determine if precueing using tactical instructions influences soccer players' decision-making skill in a sport-specific task.	13 male intermediate soccer players (19.5)) were recruited from several regional (Australia) soccer clubs.	A decision-making task that was preceded by a video containing either the basic rules of soccer (neutral condition) or specific coaching instructions that were either compatible or incompatible with the correct decision (precue condition).	Decision-making accuracy was lower and response time was faster in the precue condition.<br><br>Fixation location marginally influenced by the compatibility of the precues.<br><br>Compatible precues improve sport specific decision-making while incompatible precues hinder performance.
Bennett et al., 2019a	To investigate the construct and discriminant validity of a video-based decision-making assessment for talent identification in youth soccer.	328 male youth soccer players from three academy systems: tier one (n = 119) tier two (n = 171) and tier three players (n = 38) An additional 59 youth athletes with no competitive soccer experience in the last five years formed the control group.	Players completed a video-based decision-making assessment on an iPad, with response accuracy and response time recorded for various attacking situations (2 vs. 1, 3 vs. 1, 3 vs. 2, 4 vs. 3, and 5 vs. 3).	The video-based decision-making assessment showed construct validity.<br><br>Response times were significantly faster in the early and mid-adolescent players.<br><br>An overall decline in decision-making performance was observed from the 2 vs. 1 to the 5 vs. 3 situations.<br><br>The video-based decision-making assessment lacked discriminant validity as minimal differences between academies were evident for response accuracy and response time.

Bishop et al., 2014	Identify visual search characteristics that determined superior performance on a soccer-based task and use those characteristics in an attempt to improve novices' performance of the same task.	Experiment 1<br><br>26 male and 14 female participants, with experiences in competitive soccer ranging from complete novice to semi-professional level.<br><br>Experiment 2<br><br>A total of 46 undergraduate students who were not soccer players.	Experiment 1<br><br>96 still images depicting three oncoming attacking soccer players. Participants task was to predict the direction (left/right) in which they believed the player was about to move by clicking buttons. Eye tracking used to measures saccades.<br><br>Experiment 2<br><br>Same method, however, two groups were told to look at the head or ball as quickly as possible before making a decision.	Sole predictor of decision-making efficiency was the time taken to initiate a saccade to the ball.<br><br>There were no between- groups differences for decision-making efficiency with verbal direction.
Cardoso et al., 2021	To evaluate how response time in decision making managed by Systems 1 (intuitive) and 2 (deliberative) is associated to perceptual-cognitive processes of young soccer players.	90 male youth soccer players from the youth teams of a Brazilian First Division club.	Participants wore a mobile-eye tracking system while viewing 11-a-side match play video-based soccer simulations.	Players with shorter response time in decision making employed more fixations of shorter duration and showed less cognitive effort in the information processing, decision verbalisation and recovery phases.<br><br>The faster group also shower a greater number of thought processes associated with planning when compared to players with longer response time in decision making.
Casanova et al., 2022	To a) examine the effects induced by physiological workload on gaze behaviour during a 2 vs. 1 plus goalkeeper in-situ task and b) explore any interactions with skill level.	22 football players (defenders and/or defensive midfielders) were separated into two groups of 11 based on their tactical performance.	12 game sequences (2 vs. 1 + GK) were presented under high- and low-workload conditions. At the end of each sequence, participants indicated their perceived exertion. Visual	Football players employ different gaze behaviours during low- and high-workload conditions.<br><br>Physiological workload and tactical expertise interact to drive visual search behaviours in in-situ game situations.

			search behaviours were recorded using Tobii Pro eye-movement registration glasses.	
Caso et al., 2023	To examine whether the amount of visual exploratory activity (VEA) is differentiated within a group of elite soccer players who have been assigned specific positions in the team.	Pre-recorded video-footage of 72 players from a Dutch elite soccer club was utilised. This included 29 first team players and 32 U23 players. Each team had players proportionately distributed across positions.	VEA with respect to amount and timing for different player positions was assessed.	Compared to players positioned in the defence and attack, midfielders showed the highest engagement in VEA.<br><br>The amount of VEA during the penultimate pass predicted the success of subsequent passing actions.<br><br>The amount of late VEA, during the final pass, did not significantly add to this prediction.
Coutinho et al., 2023	To examine how the manipulation of contextual dependency and decision-making (DM) affects players' positioning and subsequent ball control, passing performance, and external load during small-sided games (SSG).	20 youth male football players from a Portuguese club competing at the regional level.	Players performed one SSG followed by one of three experimental intervention tasks: (i) low DM (ii) moderate DM and (iii) high DM. The players then performed one additional SSG bout under the same conditions.	More prescriptive tasks contributed to a higher frequency of actions but to a lower transfer to the subsequent game performance.<br><br>Adopting tasks such as the game appeared to emphasise the coupling between perception and action, thereby enhancing the players' acute response.
Dambroz et al., 2022	To compare the visual search strategies of young soccer players based on positional roles.	17, U-13 soccer players from a club in the Serie A of the Brazilian National Soccer League, grouped according to their positional roles: defenders (n= 6); midfielders (n= 6); and forwards (n= 5).	11 video scenes of offensive soccer actions, recorded and watched through a third-person perspective. Mobile eye tracking was utilised to measure visual search strategies.	Significant differences were found in total fixations in two locations: ball and teammates.<br><br>Forwards focused more frequently on the ball and teammates when compared to midfielders.<br><br>Forwards also fixated for longer periods on the ball when compared to midfielders.

de Joode et al., 2021	To establish within-group differences for game insight in elite youth soccer players; thereby, validation of the game insight indicator (GID) could be improved; (2) prospective value of perceptual–cognitive skills is examined by following the trajectory of the participants.	14 youth soccer players playing in the U12 or U13 team for an elite youth academy in the Netherlands.	The GID consisted of film clips that show game situations. Players had to predict the trajectory and destination of the ball and move toward the correct position to receive the pass of a teammate.	GID is a useful indicator for anticipating the ball's trajectory and destination at age 11–13.<br><br>No clear relationship with future performance was found,<br><br>The GID can be a valuable tool to measure and evaluate perceptual–cognitive skills to examine the process of talent development.
Eldridge et al., 2023	To understand how experienced coaches working within a professional football academy conceptualise visual exploratory activity (VEA), and how they apply that conceptualisation in their design of practice activities.	9 male football coaches working within an English Football Association Academy in the top four leagues of professional football in England.	Semi-structured interviews were conducted to gain a deeper insight into each coach's perspective of VEA.	Coaches hold VEA as an integral part of the player performance which should be coached from a young age.<br><br>Many coaches did not feel it necessary to dedicate the main focus within sessions to VEA.<br><br>Within sessions, coaches recommended delivering VEA primarily through the design of practice activities, supported by visual aids (e.g., head- bands), video review, and questioning.<br><br>Coaches desired additional evidence in support of various visual aids.
Farahani et al., 2020	To investigate the differences between Under 16, 18 and 23 academy soccer players on a video-based decision-making assessment and to compare participants' performance on the task in terms of accuracy and response time with their performance on the pitch.	73 male participants (range 15–23 years old, mean 18.6 years old).<br><br>U23 group = 26 soccer players with a mean age of 21.4. U18 group = 24 academy soccer players with a mean age of 17.8. U16 group = 23 academy	Participants watched short video clips of soccer matches - 20 trials.<br><br>Participants had to select best decision when video paused in time constrained conditions.<br><br>Coaches were asked to rate their players 1-10 for decision-	Response time was significantly lower in U23 compared to U18 and U16 and there was no significant difference between the U16 and the U18 groups, but no significant difference between age groups on the accuracy of response.<br><br>Positive correlation between accuracy on the task and general decision-making skills rated by the coaches.

		footballers with a mean age of 15.9 years.	making in attacking and defensive scenarios.	Coaches' ratings of decision-making skills and response times on the task did not correlate.
Fransen et al., 2017	To examine the influence of restricted visual feedback on the dribbling performance of youth soccer players.	189 youth soccer players aged between 10–18 years were recruited from two Belgian high level football teams. Participants were divided into three groups of fast (n = 64, average (n = 63), and slow (n = 62) dribblers.	Participants performed three assessment trials of a dribble test under different visual feedback conditions in a randomised order.	Dribbling performance was significantly impaired with decreasing visual feedback.<br><br>Decrements in dribble test performance with limited visual feedback were greater for fast dribblers when compared with slow dribblers.
Hicheur et al., 2020	To investigate if augmented feedback training can improve both perceptual–cognitive and motor skills during a passing situation in soccer.	27 elite young soccer players - males, U14 and U15 categories. They belonged to the same elite youth soccer academy and were playing at a Swiss-national level.	Three groups of players performed a test consisting in passing the ball as accurately and as quickly as possible toward a visual target moving briefly across a large screen.<br><br>The control group performed normal soccer training and was compared with two visuomotor training groups (augmented feedback and no-feedback).	Only players of the augmented feedback group significantly improved passing accuracy, reactiveness, and global passing performance, whereas the non-feedback group only improved passing accuracy. None of these parameters was improved in the control group.
Jordet et al., 2020	To investigate how professional soccer players use visual scanning in real games; establish the extent to which scanning varies under different contextual conditions; and to examine the relationships between scanning and performance.	27 professional male soccer players aged 17–32 years (M = 25.66). All players represented the same team in the EPL in the 2017/2018 season.	Players were filmed across 21 matches, producing a total number of 9,574 individual ball possessions for analysis.	Central midfielders and central defenders scan most frequently.<br><br>Less scanning is done in tight scenarios, in wide areas and closer they get to the opponent's goal.<br><br>Players scan more when they are going to pass as the next move, in particular long forward passes.

				The more a player scans, the higher probability of a successful outcome.
Keller et al., 2018	To determine if a video-based decision-making task could classify Australian older youth soccer players into different cohorts (levels of expertise) based upon their decision-making skill.	62 male youth soccer players from the Western Australian U18 state league (n = 22), WA National Training Centre (n = 22) and the Australian Institute of Sport soccer programme (n = 20) and are referred to as sub-elite, state elite and national elite, respectively.	31 clips were randomly presented for 15 s in duration and then frozen for 5 s before being occluded. Participants were required to circle on a printout of the final screenshot, the most appropriate option to pass to, or circle the goal to indicate that they would shoot at goal.	Video-based decision-making test is able to discriminate between differing levels of expertise for youth athletes.
Kent et al., 2021	To deliver and evaluate a theoretically informed performance under pressure intervention.	82 male academy soccer players.	Participants completed baseline pressure task.<br><br>Players were randomly allocated to an intervention group; receiving pressure training (PT), three cognitive behaviour workshops, and reflective diaries or comparison group; receiving PT only.<br><br>68 players repeated the PT task at a six-week follow up (B), and of these, 26 (n = 15; intervention group; n = 11; PT only) also completed a re-test PT task (A) at 12-week follow up.	Intervention players scored significantly higher in their decision-making with a significant main effect of age-group on decision-making and skill execution.
Leso et al., 2017	To identify the perception of creativity and game intelligence of soccer coaches and young players.	All athletes and soccer coaches from the 11-a-side Academic Association of Coimbra soccer team.	Questionnaire was used to investigate the perception of creativity and game intelligence of coaches and players.	There were significant differences in the importance attached to creativity and game intelligence for the players.<br><br>Coaches mostly associate the creativity in soccer to a kind of magical thinking, also

		152 participated (118 players, 34 coaches).		adding the game intelligence to the ability of rationality, problem solving, and decision- making.<br><br>There was a strong correlation between creativity and game intelligence for the players.
Machado, Cardoso & Teoldo, 2017	To assess the visual search strategy of soccer players from different age groups.	51 male youth soccer players from a Brazilian Serie A club. Players were equally grouped (n = 17) in age brackets U13, U15 and U17.	11 soccer offensive video sequences, through a third person perspective from European national championship games. Each video sequence was paused where participants had to answer “What should the player with ball possession do?”. Eye tracking glasses were used to measure visual exploratory activity.	Visual search strategy related to the number of fixations were able to discriminate U13 players from the U15 and U17 age groups.<br><br>The U15 and U17 players did not present significant differences in their visual search strategy for both number of fixation and time of fixation.
Machado et al., 2023a	To compare decision-making skills between selected and deselected players from elite Brazilian youth soccer academies.	317 Brazilian youth elite male soccer players recruited from four soccer teams that played at the first national division in Brazil (i.e. U14, U15, U16, and U17).	Players’ decision-making skills, both quality and response time, were assessed with an objective video-based test (TacticUP®).	Advantages in decision-making quality for U14 and U15 selected soccer players<br><br>Advantages in decision-making time for U14, U16, and U17 selected soccer players compared to deselected players.<br><br>Advantages of decision-making time for U15 selected players compared with those deselected.
Machado et al., 2023b	To examine the relationship between the engagement in previous developmental activities in soccer and futsal with the quality and speed of decision-making skills in different phases of sport	77 elite Brazilian professional female soccer players from six soccer teams of three different states in Brazil, competing in Brazil’s professional national league.	Players’ decision-making skills were assessed based on video-based test – TacticUP®. Retrospective questionnaire was used to collect information on participation in different developmental activities.	Engagement in deliberate practice in soccer and futsal, especially during childhood and early adolescence, is related to a better quality of offensive decision-making skills.

	development of elite female soccer players.			Engaging in deliberate play in soccer, mainly in childhood and early adolescence, is related to quicker offensive and defensive decision-making skills of elite female soccer players.
McGuckian et al., 2018	To further understanding of how exploratory head movements relate to performance with the ball, and how this relationship changes with exploration in various time-periods before gaining possession of the ball.	32 male outfield soccer players aged 16–30 years.<br><br>Participants were recruited from clubs playing in the Australian National Premier League.	Participants competed in 11v11 match-play. Outfield players wore a 9-degrees-of-freedom inertial measurement unit (IMU).	Strong relationship between head turn frequency and head turn excursion.<br><br>A higher-than-average head turn frequency and head turn excursion before receiving the ball resulted in a higher likelihood of turning with the ball, playing a pass in the attacking direction, and playing a pass to an area that is opposite to which it was received from.<br><br>When players explored their environment with higher-than-average head turn frequency and excursion, they used more complex action opportunities afforded by the surrounding environment.
McGuckian et al., 2019	1) to gain a better understanding of the exploratory actions used by footballers in a perception-action football receiving-passing task; 2) to empirically test the relationship between head movements, as a proxy for visual exploratory action, and performatory action.	12 male football players including 2 wide defenders, 4 defensive/central midfielders, 3 wide midfielders, and 3 attacking midfielders/strikers, Participants played for the same semi-professional club, competing in the Australian National Premier League.	Participants completed a simulated receiving–passing task that required them to indicate pass direction to one of four surrounding targets, as quickly as they could after they gained simulated ball possession. The frequency of head movements before and after gaining ball possession and the pass response times were recorded.	Time constraints of the task influenced the head movements and performatory actions of footballers in the passing task.<br><br>The relationship between head movements and the speed of a passing response gives further evidence for the importance of exploratory action in service of the prospective regulation of movement.
McGuckian et al., 2020	To compare the visual exploratory actions of youth	22 male outfield soccer players aged 15 to 17 years (M =	Soccer players played a total of 1,623 minutes. Inertial	Players explored more extensively when they were in possession of the ball, and

	soccer players based on; pitch location, playing role, and ball possession phase.	16.25) who had 8 to 13 years playing experience (M = 10.77).<br><br>Participants played for the same semi-professional club in the Australian National Premier League competition.	measurement units, global positioning system units and notational analysis were used to quantify relevant variables.	less extensively during transition phases, as compared to both team ball-possession and opposition ball-possession phases of play.<br><br>Players explored most extensively when in the back third of the pitch, and least when they were in the middle third of the pitch.
Meira et al., 2022	To explore visual strategies employed by female soccer players of varying expertise levels.	30 Brazilian female volunteers: 10 professional soccer players from a leading team of the Country's Major League 10 varsity soccer players, and 10 novices.	Participants were required to respond physically to film images projected on a screen using soccer skills (penalty kick, dribbling, pass reception, and defensive cover) whilst eye tracking technology examined gaze behaviour.	Female elite players engaged in more visual fixations, directed gaze toward more relevant areas of the scene, and showed less variability of pupil diameter, compared to their non-elite counterparts.
Musculus et al., 2021	To test how environmental constraints affect decision-making processes in expert and non-expert players.	80 male soccer players consisting of 40 experts (5th tier league in Germany) and 40 non-experts (two lowest German leagues).	Video based decision making tool was used to present videos with manipulated opponent pressure, time constraint and first-person perspective. Players had to make a decision at moment of video occlusion.	Players' option and decision quality improved under the time constraint but were negatively affected by opponent pressure.<br><br>The negative effects of opponent pressure were especially true under limited time and in third-person perspective.
Natsuhara et al., 2020	To analyse eye movement data and verbal reports while soccer players one-touch passed the ball in a five-on-four attacking scene.	18 high-level soccer players (HLP) and 18 middle-level soccer players (MLP).	Five-on-four soccer-specific film simulations from previously recorded real scenes, where participants reacted to the life-sized soccer scenes.	HLP group had significantly higher accuracy and consistency in decision-making compared with the MLP group.<br><br>HLPs had a significantly greater number of fixation locations than MLPs.
O'Connor et al., 2017	To investigate the pedagogical approaches coaches use to develop decision-making in soccer.	29 coaches currently working with youth soccer teams (U12, n = 5; U13, n = 8; U14, n = 4; U15, n = 4; U17, n = 8)	Youth soccer coaches were filmed conducting two practice sessions.	Strategies for incorporating decision-making into training activity – repetition of real scenarios with guided discovery, prompting decision-making by providing

		All teams competed in the Australian National Premier League News South Wales Youth competition.	The first session was a regular training session, whereas in the second session, participants were asked to create an activity they believed would promote the development of on-ball decision-making.	cues or solutions, and manipulating the game/activity.<br><br>Coaches generally use strategies to develop decision-making but generally all coaches over coach with too much stop start and instructions.
O'Connor et al., 2018	To understand how coaches conceptualise decision-making and its development in soccer.	25 participants (24 = male; 1 = female) who had a minimum of 20 years of experience in soccer.<br><br>All participants had played professional soccer in Europe and Australia for an average of 14.8 years. Since retiring from playing, all participants had become coaches.	Semi-structured interviews were carried out on participants to understand their thoughts, feelings and understanding of decision-making practices in soccer.	Participants' conceptions of good decision-makers reflected the broadening range of abilities required of players.<br><br>Participants highlighted their conceptions of how decision-making may be developed, emphasising the importance of playing with others; effective communication; balancing structure and autonomy; knowledgeable inspiration from other players and coaches; and a focus on improvement rather than winning.
Phatak & Gruber, 2019	To examine the relationship between visual exploratory frequency (VEF) and individual performance parameters in elite football midfielders.	Videos from 51 games of Euro 2016 football championships, were used where 35 male centre midfielders were used.	Video footage analysed for player scans, transition scans, and total scans.	Scanning rate of a player was positively correlated with his pass completion rate.<br><br>Scan rate while receiving the ball was negatively correlated with turnover rate.
Práxedes et al., 2018	To analyse the effect of two training programmes, each based on modified games with different level of opposition, on decision-making and technical execution in two groups of young football players of different abilities.	19 football players from the under-12 category of two teams from the same Spanish club consisting of 10 average skill and 9 low skill players.	Participants experienced 4 phases; Pre-intervention 1, Intervention 1 (teaching programme based on modified games with numerical superiority in attack), Pre-intervention 2 and Intervention 2 (teaching programme based on modified games with numerical equality). Each intervention phase lasted 14	Average skill-level group showed significant differences after the first intervention in decision-making and execution of the pass action but not after the second intervention.<br><br>For the Low skill-level group, significant differences were only observed in the execution of passing between the first and last phases.

			sessions. Decision-making and the execution of pass action during league matches over the same period were evaluated using the Game Performance Evaluation Tool (GPET).	
Pokolm et al., 2022	To model football players' scanning, integrating players' pass completion rate, and their body orientation, in relation to individual and contextual variables such as a player's appearances in national teams and the opponent pressure the player is exposed to.	8 selected matches and 11 different teams from the UEFA U17 and U19 European Championship in 2018, and 9 matches, including 12 different teams from the UEFA U17 and U21 European Championship in 2019.	Video based data analysis of the games sampled.	A player's appearances in national teams were not clearly related to body orientation and the pass completion rate.<br><br>Players who played more matches for their national team performed more scans.<br><br>Scanning had an impact on body orientation as well as on the pass completion rate.<br><br>Opponent pressure had the most substantial effect on body orientation and pass completion.
Pulling et al., 2018	To further the understanding of coaches on visual exploratory activity (VEA) and coaching practices.	303 current soccer coaches ranging in qualifications and experience.	Participants completed an online survey comprised of 3 sections and 12 items.	Those identified to have a high delivery of VEA were likely to provide more feedback/instruction on VEA; they designed an activity or part of a session to focus on VEA more often; and the percentage of sessions they would primarily focus on VEA was higher.<br><br>Higher coaching qualification and experience leads to a positive attitude of coaching VEA.
Roca et al., 2013	To examine anticipation and decision-making in skilled and less skilled soccer players.	24 adult male soccer players were recruited. Skilled participants (n = 12) were professional (n = 6) and semi-professional (n = 6) players. Less skilled players (n = 12)	Skilled and less skilled players interacted as defenders with life-size film sequences of 11 versus 11 soccer situations. Participants were presented with task conditions in which	Skilled players reported more accurate anticipation and decision-making than less skilled players, with their superior performance being underpinned by differences in task-specific search behaviours and thought processes.

		had participated only at an amateur or recreational level.	the ball was in the offensive or defensive half of the pitch.	Visual search behaviour of skilled players involved more fixations of shorter duration to significantly more locations in the visual display.
Roca, Ford & Memmert, 2018	To examine creativity in the decision making and visual search behaviours of skilled soccer players during simulated 11-a-side match play.	44 skilled, male outfield soccer players.<br><br>Players were recruited from a range of different professional and semi-professional soccer clubs in England.	Players were required to interact with a representative life-size video-based simulation of attacking situations whilst in possession of the ball. Clips were occluded at a key moment, and they were required to play the ball in response to each situation. Moreover, they were required to name other additional actions they could execute.	Creativity-based differences in judgment were underpinned by differences in visual search strategy.<br><br>Most-creative players employed a broader attentional focus including more fixations of shorter duration and towards more informative locations of the display compared with least-creative players. Most-creative players detected teammates in threatening positions earlier in the attacking play.
Roca & Ford, 2020	To investigate the types of practice activities used by coaches of elite youth players across European countries.	53 male soccer coaches working with boys under 12 to under-16 age groups within 16 Youth Academy professional top-division clubs of four European nations.	83 practice sessions from under-12 to under-16 age groups was assessed - around 20 from each country for four clubs.	More time was spent in active decision-making compared to non-active decision-making activities and transitioning between activities.<br><br>Portugal and Spain spent a higher amount of time in active decision-making activities compared to English and German players.<br><br>English players spent more time in unopposed technical-based drills and German players in improving fitness aspects of the game without the ball.
Roca, Ford & Memmert, 2021	To determine the underlying perceptual and cognitive processes that underpin creative expert performance in the sport of soccer.	40 male outfield soccer players took part in the study. These players were recruited from a range of different professional	Participants interacted with representative video-based 11 vs. 11 attacking situations whilst in possession of a ball. Clips were occluded at a key	High-creative players made more fixations of shorter duration in a different sequential order and to more task-relevant locations of the display.

		and semi-professional soccer clubs in Southeast England.	moment and participants were required to play the ball in response to each presented scenario.<br><br>Moreover, they were required to name other additional actions they could execute for each situation.	High creative players generated a greater number of verbal reports of thoughts related to the assessment of the current task situation and planning of future decisions when compared with the low-creative players.
Savelsbergh et al., 2010	To examine whether differences in visual search and locomotion behaviour can be identified in a homogenous group of young soccer players who were selected as talented soccer players.	20 male players, age 10-13 years. All participants played for the regional selection team of the Royal Dutch Soccer Association or for a professional club.	Participants watched video clips of a 4-to-4 position game.<br><br>Participants were asked to take part in the game by choosing the best position for the reception of the ball passed.	The high-score group looked more to the ball area, while the players in the low-score group concentrated on the receiving player and on the hips/upper-body region of the passing player.<br><br>The high-score group was better in moving to the correct direction and to the correct area to receive the ball than the low-score group.
Teoldo et al., 2023	1) To compare the number of decisions made by soccer players at academy and professional levels in official matches; 2) compare the quality and speed of decision-making between academy and professional soccer players.	Study 1 - 24 soccer teams, from which 14 were professional teams that played in the 2012/2013 UEFA Champions League, whereas 10 were U-20 academy teams that played national tournaments in Brazil.<br><br>Study 2 - 138 male soccer players, from which 42 were professional and 96 were young academy players (62 U17 and 34 U20 players).	Study 1 – Notational analysis of 12 official matches<br><br>Study 2- TacticUP® was used to assess game reading and decision- making skills.	Professional players made more decisions in official matches when compared to academy players.<br><br>Professional players made quicker decisions than academy players in offensive and defensive actions, inside and outside the centre of play, and both with and without the ball.
Vaeyens et al., 2007a	To examine differences in decision-making skill and visual search strategies across five categories of small-sided,	87 male adolescents. The elite group consisted of 21 youth players. The sub-elite group	Film simulations of offensive play, utilising movement-based response measures, and an eye movement registration.	Playing experience, skill level, and the unique constraints of the task, expressed by the number of players and relative proportion of offensive and defensive

	offensive game simulations in soccer.	included 21. The third subgroup consisted of 23 soccer players.		players, determined both the observed search behaviour and processing requirements imposed on players in dynamic offensive team simulations.
van Maarseveen et al., 2018	To examine how well performance in a complex time-constrained motor task could be predicted using representative tests of perceptual- cognitive skill.	22 highly talented female soccer players from the national soccer talent team participated in the study.	3v3 performance analysed by coaches before participants completed lab based, video interactions of 3v3 recordings.	On-field performance could not be predicted on the basis of performance on the perceptual-cognitive tests.<br><br>There were no strong correlations between the level of performance on the different tests.<br><br>Perceptual-cognitive tests may not be as strong determinants of actual performance as may have previously been assumed.
Vater, Luginbühl, & Magnaguagno, 2019	To investigate perception-action coupling of defenders in 3 vs. 3 defensive situations and combine an on-field decision making test with verbal reports to examine how expert soccer players use their peripheral vision.	10 high-skilled and ten low skilled male soccer players.	Mixed-methods approach with in-situ decision making (3 vs. 3 situations) and retrospective verbal reports to identify perceptual strategies used for optimal information pick-up in high- and low-skilled soccer players.	High skilled players seem to actively monitor players' actions with peripheral vision, while low skilled players rely more on bottom-up information changes that are perceived with peripheral vision.<br><br>The use of peripheral vision by central defenders depends on the position of the ball and the position of the direct opponent.
Vítor de Assis et al., 2020	To compare the decision-making and visual search strategies of young soccer players between two groups based on the results on-field specific tactical test.	90, U-15 male soccer players affiliated to three regional Brazilian clubs.	Tactical behaviour was assessed using the FUT-SAT assessment tool.<br><br>Video-based tests were used to assess decision-making, while visual search strategies were performed.	More skilled participants showed better decision-making scores compared to the less skilled.<br><br>The more skilled performed a higher mean number of fixations per trial and had a lower mean duration of fixation.

Vítor de Assis et al., 2021	To compare the visual search strategy and anticipation between two groups of young players of different efficiencies in tactical behaviour.	44 Brazilian male soccer players from three regional clubs.	Tactical behaviour was assessed using FUT-SAT; anticipation score was obtained by a video-based assessment, while visual search strategy was performed using the Mobile eye tracking.	There seems to be an interaction between cognitive skills such as anticipation and visual search strategy with the application of tactical behaviour by soccer players.<br><br>The more tactically efficient soccer players showed a different visual search strategy and better anticipation judgments.
Ward & Williams, 2003	To examine how visual, perceptual, and cognitive skills develop as a function of age and skill in soccer.	Elite and sub-elite male soccer players (N = 137) were selected as participants.<br><br>Elite players were recruited from English Premier League Academies, while sub-elite players were recruited from local elementary and secondary schools.	Multidimensional battery of tests utilised to measure static visual acuity; dynamic visual acuity; stereoscopic depth sensitivity; and peripheral awareness.	The tests of visual function did not consistently discriminate between skill groups at any age.<br><br>Tests of anticipatory performance and use of situational probabilities were the best in discriminating across skill groups.<br><br>Memory recall of structured patterns of play was most predictive of age.<br><br>As early as age 9, elite soccer players demonstrated superior perceptual and cognitive skills when compared to their sub-elite counterparts.

Table 4. Theme analysis summary.

Theme Identified	Sub-Theme	Study Key Words		
Perceptual cognitive skills	Visual search behaviours	• Anchor<br>• Exploration<br>• Exploratory activity<br>• Eye movements<br>• Gaze behaviour	• Gaze strategies<br>• Orientation<br>• Pivot<br>• Scans<br>• Scan frequency<br>• Scanning<br>• Vision	• Visual attention<br>• Visual exploratory activity<br>• visual exploratory frequency<br>• Visual function<br>• Visual perception<br>• Visual search<br>• Visual search strategy
Perceptual cognitive skills	Cognitive capabilities	• Affordance<br>• Anticipation<br>• Attention<br>• Awareness<br>• Cognition<br>• Cognitive effort<br>• Decision-making	• Game intelligence<br>• Locomotion behaviour<br>• Option generation<br>• Pattern recall<br>• Perception<br>• Perception–action<br>• Perceptual cognitive expertise	• Situation awareness<br>• Situational probabilities<br>• Spatial<br>• Tactical awareness<br>• Tactical knowledge<br>• Take-the-first heuristic<br>• Transition scans
Performance		• Ball control<br>• Expertise<br>• Game reading<br>• Motor control<br>• Motor skill<br>• Performance	• Expertise and expert performance<br>• Perceptual-motor skills<br>• Passing percentage	• In situ performance<br>• Passing behaviour<br>• Passing performance<br>• Response time<br>• Sports performance<br>• Tactical performance<br>• Technical performance<br>• Turnovers
Methodologies/interventions		• Assessment<br>• Augmented feedback training<br>• Behaviour<br>• Constraints<br>• Ecological psychology	• Performance intervention<br>• Playing position<br>• Practice activities<br>• Precues<br>• Representative design	• Representative learning design<br>• Situational task constraints<br>• Stroboscopic vision<br>• Systematic observation<br>• Task constraints

		• Eye-tracking<br>• Eye movements<br>• Feedback<br>• Game insight indicator<br>• Match analysis<br>• Match play<br>• Occlusion<br>• Occlusion task<br>• Performance analysis		• Task representative<br>• Verbal reports
Methodologies/interventions	Sport context/participants	• Adolescence<br>• Athletes<br>• Coaching<br>• Deliberate practice/play and futsal<br>• Elite athletes<br>• Elite youth<br>• Development<br>• Female football	• Skill acquisition<br>• Small-sided games<br>• Soccer<br>• Sport<br>• Football<br>• Pedagogy<br>• Practice design<br>• Team sports	• Talent development<br>• Talent identification<br>• Talent identification and selection<br>• Training tasks<br>• Women<br>• Youth<br>• Youth Sports<br>• Youth elite soccer players
Miscellaneous		• Coping<br>• Engagement<br>• Learning<br>• Mediating mechanisms	• Mental toughness<br>• Pass receive<br>• Player-role<br>• Resilience<br>• Skill<br>• Tactics	• Yo-Yo test

i

888

Figures

Click here to access/download;Figure;PCS Figures 2024 Submission.docx

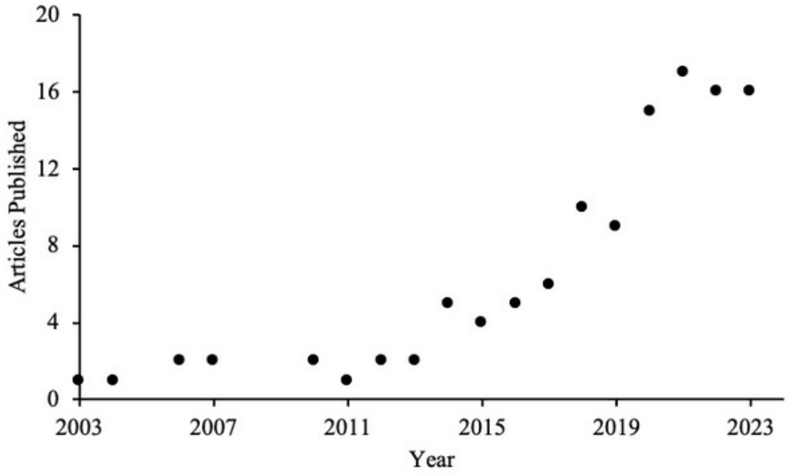

Figure 1. Number of PCS publications returned from initial inclusion criteria search.

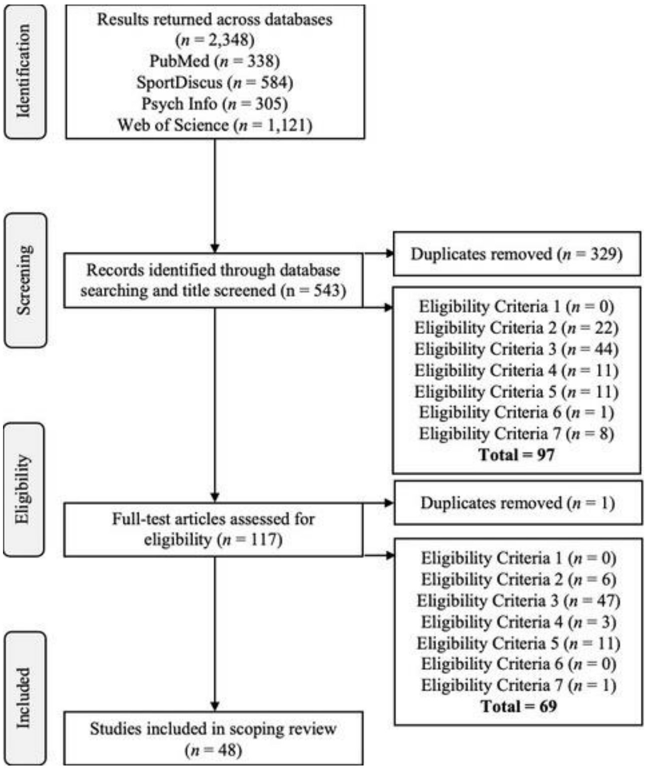

Figure 1. *PRISMA flow chart of process to generate studies for review.*

Figure 1. PRISMA Flow Chart

Click here to access/download;Figure;Figure 1. PRISMA Flow Chart.docx

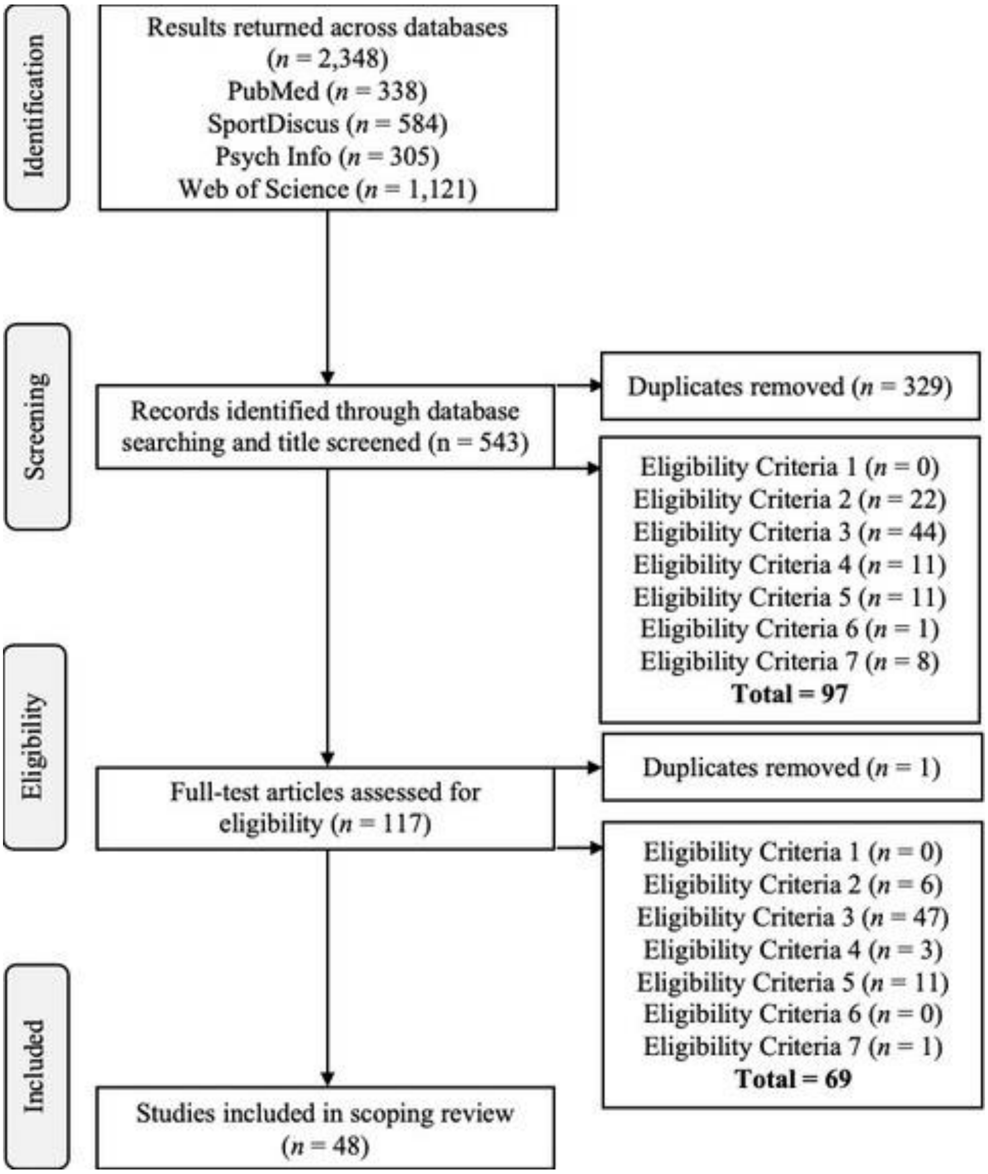

***Figure 1.** *PRISMA flow chart of process to generate studies for review.*
