# Supplementary material for: Perceptual-cognitive skills and talent development environments in soccer: A scoping review
Source: PLoS One. 2025 Jul 8;20(7):e0327721. doi: 10.1371/journal.pone.0327721 (PMC12237070; doi:10.1371/journal.pone.0327721)
Supplement: Attachment — Submitted filename: Reponse to Reviewers PONE-D-24-10341.docx [file pone.0327721.s003.docx]

UCFB Manchester Campus,

14th Floor, 111 Piccadilly, Manchester, M1 2HY,

United Kingdom

[a.o.triggs@2021.ljmu.ac.uk](mailto:a.o.triggs@2021.ljmu.ac.uk)

Dear Dr Andreato,

Thank you for the valuable feedback and insightful comments provided by the reviewer on our manuscript entitled “Perceptual-cognitive skills and talent identification in soccer: A scoping review of the last 20 years” (Manuscript ID: PONE-D-24-10341). We appreciate the time and effort the reviewer has taken to assess our work, and we are grateful for the opportunity to revise and improve our submission.

We have carefully considered and addressed all the comments and suggestions provided. Below, we outline the changes made in response to each comment made by the reviewer. We believe that these revisions have strengthened the manuscript significantly. Please find resubmitted our revised manuscript: “**Perceptual-cognitive skills and talent development environments in soccer: A scoping review**”

The resubmission has been approved by all authors, and if accepted, it will not be published elsewhere.

**Reviewer #1**

**Comment** 1: Re-word doesn't make sense (line 23-25).

**Response**: We thank the reviewer for highlighting this issue. In response, we have re-worded the abstract introduction. This change can be found in line 1-5.

**Comment** 2: Depends on how you define performance (line 23).

**Response**: We appreciate the reviewer’s feedback on this point. We maintain that perceptual-cognitive skills are a required component of performance within professional soccer and have re-phrased to capture this more suitably. This change can be found on line 1.

**Comment** 3: - used above. Be consistent (line 26)

**Response**: We appreciate the reviewer’s feedback on this point. We have now made sure perceptual-cognitive skills maintain the use of the hyphen whenever the term is written in full throughout the manuscript.

**Comment** 4: For richer clubs I would add big data and machine learning (line 41-44).

**Response**: We appreciate the reviewer’s feedback on this point. As suggested, we have aimed to capture the incorporation of data science within talent programmes. This addition can be found on line 21.

**Comment** 5: This is too vague and superficial. Re-word and summarise what you think is the culmative evidence of the work above (line 52-54)

**Response**: We thank the reviewer for highlighting this issue. As suggested, we have re-framed this paragraph to highlight the breadth of research surrounding talent development environments with a lack of research to show it’s applied impact and the use of evidence-based principles/frameworks. The revised text is now located in line 28-33.

**Comment** 6: I would lose this. I don't think you need to go down this rabbit hole. I would get straight into the PCS debate (line 55-66).

**Response**: We agree with the reviewers comments and have removed this paragraph as a result, moving straight into the PCS component of the introduction.

**Comment** 7: In view of my earlier suggestion this needs to be 'beefed up' and more relevant to TID/TDE processes/outcomes. I also need to confirm at this stage the focus will be academy/professional settings. I am reading words such as 'expert', 'performance' etc.. (line 67-82).

**Response**: We appreciate the reviewer’s feedback on this point. We have modified this paragraph to provide a clearer overview of PCS impact and importance within soccer and how it can support them moving towards future expert performance. The link with TDE is hopefully now clearer, where we have highlighted the need for practitioners to identify, assess and develop these skills to support players with the required skills for first team football. Although expert and performance terms are still incorporated, this is only referring to how we know PCS support future expert performers, and this is why TDE practitioners should invest in supporting athletes with these skills. This change can be found in line 42-55.

**Comment** 9: This confirms my point above. This is not supported and too vague. (what does detailing the manner these skills...mean) (line 84-86).

**Response**: We appreciate the reviewer’s feedback on this point. We have re-framed the review more specifically around TDE and PCS (as opposed to TID). And we have tried to be more specific around what the review will provide, whilst also accepting the purpose scoping reviews have in providing an overview of the current literature. The revised text is now located in line 67-75.

**Comment** 10: Interesting I would have though you have enough of a rationale for a full PRISMA SR and a quality appraisal etc...but will read on (line 87-89)

**Response**: We appreciate the reviewer’s feedback on this point. We do however maintain our position and rationale for a scoping review, which we have tried to be clearer on throughout the paper.

**Comment** 11: In what? (line 91)

**Response**: We appreciate the reviewer’s highlighting this. We have made clear it is PCS research in soccer specific environments. This amendment can be seen in line 79.

**Comment** 12: A couple of things here. Just because this is the first of something doesn’t mean it's needed. This is language games we adopt because university admin who write REF impact cases ask us to use this language.

Second - I thought this review was around PCS and TID/TDE it now suggests soccer-specific environments (so does now include first team etc..?)

Third - The introduction is problematic for me at the moment. The absence of a clearly delineated and defined RQ or RQ's makes it difficult to establish what the MO for the review is. (line 93)

**Response**: We appreciate the reviewer’s feedback here. Regarding point one, we maintain this review is one of the first to explore PCS, soccer and links to TDE. However, hopefully the re-framing of the introduction now makes it clearer as to its need. Regarding point two, the review sought literature in soccer that had findings that could be applied to TDE environments/applied practitioners. For example, if there is research around how frequent players scan, then that paper may provide applications for practitioners working with players (exploratory nature of ScR). However, we have now made it more specific around TDE than TID. Thirdly, we agree the research question should have been introduced earlier, and so have brought it forward to line 81.

**Comment** 13: I wonder whether you could have achieved this with a full SR. A narrative analysis a quality appraisal would have given you a nice discussion. Not a deal-breaker for me but it just seems a little strange (line 98-100).

**Response**: We appreciate the comment raised. We maintain a scoping review was appropriate due to diverse methodologies even post inclusion/exclusion criteria rating and the aim to provide an overview of the PCS, soccer, TDE links and where researchers should look to further support applied practices. We have aimed to be more consistent with this rationale throughout and can be seen in line 89-95.

**Comment** 14: Which isn't stated (line 102)

**Response**: We appreciate the reviewer’s feedback on this point. As suggested, we brought the research question forward (line 81).

**Comment** 15: Ah...the RQ. This needs to go earlier after the introduction. This is different to the aim/objective presented earlier. This is a deal-breaker. What do you mean by TID/TDE outcome focused. This is vague and superficial and needs to be more precise. What type of soccer-specific environments (academy, schools, grassroot clubs, regional camps, university, college...I could go on). This needs to be delineated or defined. From reading the introduction you refer specifically to academy setting (Anglophile) - this seems much broader. Again I could be wrong. Your job is the give me precise information (line 105-107).

**Response**: We appreciate the reviewer’s concern on this point. As suggested, we have aimed to make the research question and focus of the review clearer, moved more specifically to TDE (than TID) and have aimed to make it clearer what we mean by this and the purpose of the review. These changes and refined clarity have been introduced in line 68-75.

**Comment** 16: Why 20 years. What's the rationale? Was this when a seminal PCS paper was published? (line 107).

**Response**: We appreciate the reviewer’s comment here. This was a review of the last 20 years due to the increased research interest in PCS since 2000 (Figure 1). However due to the time this paper was in holding, we have re-framed to examine 3 decades of evolving enquiry into PCS and soccer (line 78-81).

**Comment** 16: Not your fault but this was approaching four months ago. Can you re-run and establish whether any new articles could be added (line 109).

**Response**: We appreciate the reviewer’s comment here. We have re-run searches here to update to January 2025 and modified the report accordingly (line 101).

**Comment** 17: Any journal citation chaining used as a back stop? (line 111-112).

**Response**: We appreciate the reviewer’s feedback on this point. Although this would have been a sensible approach, we believe the coverage from four databases with additional handpicking process from google scholar help provide adequate coverage of relevant articles. Upon reflection, this is something that could have been included but has not been evidenced by other scoping reviews on similar areas (e.g., McCalman et al., 2021).

**Comment** 18: This alludes to the earlier point about the Introduction. If this is about PCS in a TID/TDE context then I would expect a more comprehensive argument to be presented (line 115-116).

**Response**: We appreciate the reviewer’s feedback on this point. We have included a clearer rationale for the inclusion of the ‘coach*’ search term in line 109-110.

**Comment** 19: If this review is about PCS and TID how come scouts was not included as a search term? (line 116).

**Response**: We appreciate the reviewer’s concern here. Following a re-framing around TDE, the rationale for incorporating scouts in this in now no longer required.

**Comment** 20: Wow. Enough for a SR.

**Response**: We appreciate the reviewer’s feedback here. After reviewing recent scoping reviews of a similar theme, we feel the number of search terms were in-line with other scoping review (e.g McCalman et al., 2021).

**Comment** 21: These are really vague. So I assume you are including literally all environments. There is no mention of gender (so assume male and female environments). I am still not sure I understand what applications/implications actually means? So if there is a coach perception of PCS in a talent setting this will be included. Does stakeholders extend to parents/teachers? These are not included in the search parameters and important if the environment is a school, college or university for example. How or where does the TID/TDE application appear in the study. Does the MO for the inclusion criteria mean a study must outline in the aim/objective TID/TDE? This is not clear. (line 132-136).

**Response**: We appreciate the reviewer’s feedback on these points. For point 1, we have aimed to be clearer on the inclusion criteria and rationale for this. Please see line 125-132. For point 2, we hope we have now made the approach to research applications clearer and can be seen in line 67-70. For point 3, line 127-130 highlight how the TDE link was searched for during screening.

**Comment** 22: In your final included studies you have a population that includes GK's does this not violate your inclusion criteria? (line 136)

**Response**: We appreciate the reviewer’s comment. We believe the framing of our criteria is suitable as the use of the goalkeeper in the study referred to is not the focus of the paper. The studies had to focus on outfield players and not include penalty kick analysis (line 131).

**Comment** 23: How was this resolved if JC and AM did not agree? (Line 142-144).

**Response**: We appreciate the reviewer’s feedback on this point. This sentence has been amended, reflected in line 139-140.

**Comment** 24: What data? Quants/Quals. Did you have a codebook to extract data and adopt another strategy? What are recordings? Can you again be precise with language (line 144-145).

**Response**: We appreciate the reviewer’s comment. We have re-framed some of the language used alongside introducing an analysis section to be clearer on how the papers were analysed (line 140-166).

**Comment** 25: What quality checks? Assume you don't mean quality appraisal here? (line 147)

**Response**: We appreciate the reviewer’s feedback on this point. We did not mean quality appraisal, and were referring to members of the research team reviewing articles selected/deselected to ensure the criteria set was being used appropriately.

**Comment** 26: Which is not clear (for me at least) (line 149).

**Response**: We appreciate the reviewer’s comment on this point. The data analysis section has been re-written and can be seen on line 148-166.

**Comment** 27: So are you only extracting quantitative data to establish associations between different variables? (line 150)

**Response**: We appreciate the reviewer’s comment on this point. This section has been re-written and can be seen in line 154-166.

**Comment** 28: Sorry to sound like a stuck record but again this is really vague. It doesn't really tell me anything. (line 150-152)

**Response**: We appreciate the reviewer’s comment on this point. This section has been re-written and can be seen in line 154-166.

**Comment** 29: And this for me is problematic. Even though it is a PRISMA ScR - it needs to be clearly defined (see Boland, Cherry and Dickson). I accept it can be broader in focus but it still requires a clearly defined question. If you maintain this position I would suggest you change the MO to a 'narrative review' rather than a PRISMA-ScR. (line 156).

**Response**: We thank the reviewer’s for their feedback. Line 146-150 continue to develop the rationale for the scoping review, alongside a more clearly defined research question than previous.

**Comment** 30: Was this decided post-hoc? (line 157)

**Response**: We appreciate the reviewer’s feedback here. Analysis of methodological approach quality was not undertaken with it not being a key feature of ScR.

**Comment** 31: This is not mentioned. This is another deal-breaker for me. What was the analysis? This needs to be outlined? (line 166)

**Response**: We appreciate the reviewer’s concern here. We have addressed this with the inclusion of an analysis section within the methodology (line 154-166).

**Comment** 32: Where did this theme originate form? Did you have a codebook where you deductively extracted a prior items/data? (line 171)

**Response**: We appreciate the reviewer’s feedback here. Hopefully the inclusion of the analysis section and more specific methods highlights how themes were generated (line 154-166).

**Comment** 33: Considering the MO for this review is PCS in TID/TDE there is not discussion around this here. I am not sure anyone working in TID/TDE would gain anything from reading this. (line 173-193)

**Response**: We appreciate the reviewer’s concern here. We have re-framed significant portions of the manuscript to reflect more specific links with TDE throughout.

**Comment** 34: It would be useful to *those included studies which you are referring to. (line 156)

**Response**: We appreciate the reviewer’s feedback on this point. As suggested, reviewed studies have been asterisked throughout.

**Comment** 35: You evidence from Australian youth academies above ob l.197 (line 201)

**Response**: We acknowledge the reviewer’s point here. We have re-written this paragraph to acknowledge the need for visual search research in younger age groups within TDE environments and can be reflected in line 212-229.

**Comment** 36: This has lurched to UK/Anglophile terminology. An audience outside of the UK/England will not know what youth development phase means. You have gone very specific to academy settings (assume in England). You seem to be introducing ideas in here that the review is not supporitng? Why not extend the point to other contexts? (line 201-205)

**Response**: We appreciate the reviewer’s feedback on this point. As suggested, we have moved the language award from UK terminology to support practitioners in a broader sense (e.g., line 221-224).

**Comment** 37: Wow. This is quite a leap. I am not sure you have the evidence to make this claim yet. It certainly will not help guide practitioners. Be specific. How will this guide practitioners? (line 205-207)

**Response**: We appreciate the reviewer’s feedback here. We agree with the comment and have removed the statement in question and re-written, focusing on the importance of understanding scanning abilities at younger ages.

**Comment** 38: Okay how does link to TID/TDE (line 323-335).

**Response**: We appreciate the reviewer’s point here. We have re-written this paragraph to try and provide consistent links to TDE throughout the discussion and can be seen in line 349-363.

**Comment** 39: Probably more with the TDE in this context (line 378)

**Response**: We appreciate the reviewer’s feedback on this point. We agree coaches are more TDE oriented and have modified this section to reflect this (line 413).

**Comment** 40: Do university degrees such as SES and Sport Coaching at both BSc or MSc not do this? Or is this not what you mean? (line 379-380)

**Response**: We appreciate the point raised. We were referring to the practitioners who ID and develop PCS, referring to coaches in this instance.

**Comment** 41: Why this region only? (line 382-383)

**Response**: We appreciate the reviewer’s feedback on this point. This sentence has been re-framed as to not take away from the point this section was trying to make, line 418-419.

**Comment** 42: Reword - this is not clear (line 385-386)

**Response**: We appreciate the reviewer’s comment. This sentence has been re-worded (line 418-421).

**Comment** 43: Apologies, but again I do not know what you mean here. Can you re-phrase of add an example of what you mean (line 387-388).

**Response**: We appreciate the comment here. Line 421-424 reflect the re-phrasing of this section.

**Comment** 44: Ditto above. Re-word for clarity. (line 389-390)

**Response**: We appreciate the reviewer’s feedback on this point. Line 425-431 reflect the re-wording for enhanced clarity.

**Comment** 45: So what are you suggesting? Are you suggesting coaches need training in PCS or to work alongside PCS experts? Again this is not clear (line 391-393)

**Response**: We appreciate the reviewer’s feedback here. In this section we are referring to the point that due to coaches role in the assessment and development of PCS, we should have a better understanding of how their knowledge and practices align with PCS assessment, monitoring and development. This has been re-worded in line 426-432.

**Comment** 46: Of what? Perception of PCS? What practices? Do you mean coach practice? What knowledge and action? (line 394)

**Response**: We appreciate the reviewer’s comment on this point. This has been re-worded for improved clarity (line 428-432).

**Comment** 47: Not just at youth level. Domestic, technical and lead scouts also identify potential first-team players (line 400).

**Response**: We appreciate the reviewer’s feedback here. We have re-framed for scouts in TID contexts (line 434).

**Comment** 48: What does 'traditional' mean? Do you mean sitting in a stand? Or do you mean using technology such as Wyscout? (line 401).

**Response**: We appreciate the reviewer’s comment. We have aimed to be clearer on what we mean by traditional scouting approaches (line 435-437).

**Comment** 49: Re-word (line 410)

**Response**: We appreciate the reviewer’s feedback on this point. As suggested, we have re-worded this section (line 444).

**Comment** 50: Are you discussing studies from the review or adding in additional evidence here? (line 418-420)

**Response**: We appreciate the reviewer’s comment on this point. Now reviewed studies contain an asterisk, this should be clearer on the evidence used (line 461).

**Comment** 51: Does this not need a REF? (line 418)

**Response**: We appreciate the reviewer’s feedback here. This section has been re-written and so this sentence is no longer in the text.

**Comment** 52: This section needs to hammer home how this review recorded a dearth of PCS studies in female populations and how we need to avoid replicating previous PCS studies from male contexts. (line 425-433)

**Response**: We appreciate the reviewer’s feedback on this point. As suggested, we have re-written and amended this section to emphasise the importance of females specific PCS-soccer research (line470-486).

**Comment** 53: Goes back to my earlier point. Why this wasn't a full SR with a quality appraisal would have made this process (probably longer) but easier in the long run. (line 449-450)

**Response**: We appreciate the reviewer’s feedback here. We maintain our position on the ScR, to provide an overview of the research done an identify areas to further support the applied field.

**Comment** 54: This is where the absence of a clearly defined RQ is causing you a problem. These are vague and fluffy and I have no idea what they mean. (line 451-454)

**Response**: We appreciate the reviewer’s feedback on this point. As suggested, we have [explain the changes made]. We believe this revision improves [clarity, precision, etc.] in the manuscript. The revised text is now located in [section or page number].

**Comment** 55: Point one - who you mean by key stakeholders? Surely they are already integrated in a TID/TDE context. (line 455-456)

**Response**: We appreciate the reviewer’s feedback on this point. The recommendations section has been re-written and can be seen in line 500-530.

**Comment** 56: Such as? Provide an example (line 475-458)

**Response**: We appreciate the reviewer’s feedback on this point. The recommendations section has been re-written and can be seen in line 500-530.

**Comment** 57: This is a really disappointing ending. There is nothing in the conclusion that moves this world forward. There are too many vague and speculative suggestions not supported in the review. This section needs a re-write. (line 461)

**Response**: We appreciate the reviewer’s feedback on the conclusion. As suggested, we have re-written the conclusion to be more specific around PCS, soccer and TDE.

**Comment** 58: What are the original contributions?. (line 462-463)

**Response**: We appreciate the reviewer’s feedback on this point. The contributions are the comprehensive overview of the current state of knowledge in soccer-specific contexts, line 534.

**Comment** 59: First time this analysis approach is mentioned (line 465-466)

**Response**: We appreciate the reviewer’s comment. Analysis section included now should support the conclusion.

**Comment** 60: But you included studies outside of the youth age range. Are you accepting 23 years as youth? (line 466).

**Response**: We appreciate the reviewer’s feedback on this point. The rationale in the methods for why studies with older participants were included should support the comment made here.

**Comment** 61: Re-word this is too vague and superficial. This is a theme running through this review. There needs to be more specific rather than vague statements. (line 468-469)

**Response**: We appreciate the reviewer’s feedback here. As suggested, we have aimed to be more specific throughout the review and within the conclusion (line 533-546).

**Comment** 62: Such as? What gaps did this review establish? (line 468)

**Response**: We appreciate the reviewer’s feedback on this point. The gaps are re-iterated in the future directions alongside a secondary reference to them in the conclusion (line 533-546)

**Comment** 63: Is this not a statement of the obvious. Did you need to conduct a review to establish this. Again, be specific. Nail you colours to the mast. (line 470-471)

**Response**: We appreciate the reviewer’s feedback on this point. We hope the significant amendments to the manuscript reflect the need for this review and the areas that need addressing the PCS-soccer domain in order to support TDE environments and their practitioners (line 533-546).

**General Revision and Additional Changes:**

In addition to the specific responses above, we have made some further revisions to improve the clarity and quality of the manuscript. These include a more specific focus on talent development environments and its practitioners alongside a more specific rationale for the scoping review.

We believe these revisions have addressed the reviewer’s concerns and have substantially improved the manuscript. We sincerely hope that the changes meet your expectations, and we are happy to provide further clarifications or revisions if needed.

Thank you once again for your time and consideration. We look forward to your feedback and hope that the revised manuscript will now be suitable for publication in PLOS One.

Yours sincerely,

Andrew Triggs

Lecturer

UCFB

[a.o.triggs@2021.ljmu.ac.uk](mailto:a.o.triggs@2021.ljmu.ac.uk)
